# The spectrum of association in HLA region with rheumatoid arthritis in a diverse Asian population: evidence from the MyEIRA case-control study

**DOI:** 10.1186/s13075-021-02431-z

**Published:** 2021-01-30

**Authors:** Lay Kim Tan, Chun Lai Too, Lina Marcela Diaz-Gallo, Sulaiman Wahinuddin, Ing Soo Lau, Hussein Heselynn, Shahril Nor-Shuhaila, Suk Chyn Gun, Mageswaran Eashwary, Mohamed Said Mohd-Shahrir, Mohd Mokhtar Ainon, Rosman Azmillah, Othman Muhaini, Murad Shahnaz, Lars Alfredsson, Lars Klareskog, Leonid Padyukov

**Affiliations:** 1 Immunogenetic Unit, Allergy and Immunology Research Center, Ministry of Health Malaysia, Institute for Medical Research, National Institutes of Health Complex, Shah Alam, Selangor Malaysia; 2 Faculty of Medicine, Universiti Kuala Lumpur Royal College of Medicine Perak, Ipoh, Perak Malaysia; 3 Department of Medicine, Division of Rheumatology, Karolinska Institutet and Karolinska University Hospital, Stockholm, Sweden; 4 Department of Medicine, Ministry of Health Malaysia, Hospital Raja Perempuan Bainun, Ipoh, Perak Malaysia; 5 Department of Medicine, Ministry of Health Malaysia, Selayang Hospital, Selayang, Selangor Malaysia; 6 Department of Medicine, Ministry of Health Malaysia, Putrajaya Hospital, Putrajaya, Malaysia; 7 Department of Medicine, Ministry of Health Malaysia, Hospital Tuanku Ja’afar Seremban, Seremban, Negeri Sembilan Malaysia; 8 Faculty of Medicine, Universiti Kebangsaan Malaysia Medical Center, Kuala Lumpur, Malaysia; 9 Department of Medicine, Ministry of Health Malaysia, Tengku Ampuan Afzan Hospital, Kuantan, Pahang Malaysia; 10 Ministry of Health Malaysia, Federal Government Administrative Center, Putrajaya, Malaysia; 11 Institute of Environmental Medicine, Karolinska Institutet, Stockholm, Sweden

**Keywords:** Rheumatoid arthritis, HLA amino acid residues, Risk variants, HLA fine-mapping, Multi-ethnic Malaysian population

## Abstract

**Background:**

Fine-mapping of human leukocyte antigen (*HLA*) region for rheumatoid arthritis (RA) risk factors has identified several HLA alleles and its corresponding amino acid residues as independent signals (i.e., *HLA-A*, *HLA-B*, *HLA-DPB1*, and *HLA-DQA1* genes), in addition to the well-established genetic factor in HLA-DRB1 gene. However, this was mainly performed in the Caucasian and East Asian populations, and data from different Asian regions is less represented. We aimed to evaluate whether there are independent RA risk variants in both anti-citrullinated protein antibody (ACPA)-positive and ACPA-negative RA patients from the multi-ethnic Malaysian population, using the fine-mapping of *HLA* region strategy.

**Methods:**

We imputed the classical *HLA* alleles, amino acids, and haplotypes using the Immunochip genotyping data of 1260 RA cases (i.e., 530 Malays, 259 Chinese, 412 Indians, and 59 mixed ethnicities) and 1571 controls (i.e., 981 Malays, 205 Chinese, 297 Indians, and 87 mixed ethnicities) from the Malaysian Epidemiological Investigation of Rheumatoid Arthritis (MyEIRA) population-based case-control study. Stepwise logistic regression was performed to identify the independent genetic risk factors for RA within the HLA region.

**Results:**

We confirmed that the HLA-DRB1 amino acid at position 11 with valine residue conferred the strongest risk effect for ACPA-positive RA (OR = 4.26, 95% CI = 3.30–5.49, *P*_*GWAS*_ = 7.22 × 10^−29^) in the Malays. Our study also revealed that HLA-DRB1 amino acid at position 96 with histidine residue was negatively associated with the risk of developing ACPA-positive RA in the Indians (OR = 0.48, 95% CI = 0.37–0.62, *P*_*GWAS*_ = 2.58 × 10^−08^). Interestingly, we observed that HLA-DQB1*03:02 allele was inversely related to the risk of developing ACPA-positive RA in the Malays (OR = 0.17, 95% CI = 0.09–0.30, P_*GWAS*_ = 1.60 × 10^−09^). No association was observed between the HLA variants and risk of developing ACPA-negative RA in any of the three major ethnic groups in Malaysia.

**Conclusions:**

Our results demonstrate that the RA-associated genetic factors in the multi-ethnic Malaysian population are similar to those in the Caucasian population, despite significant differences in the genetic architecture of *HLA* region across populations. A novel and distinct independent association between the HLA-DQB1*03:02 allele and ACPA-positive RA was observed in the Malays. In common with the Caucasian population, there is little risk from HLA region for ACPA-negative RA.

**Supplementary Information:**

The online version contains supplementary material available at 10.1186/s13075-021-02431-z.

## Background

Extensive genetic studies during the last 40 years have demonstrated substantial contribution from the human leukocyte antigen DR beta chain 1 shared epitope (*HLA-DRB1* SE) alleles in RA pathogenesis, specifically for the subtype of RA that is positive for anti-citrullinated protein antibody (ACPA) [1–11]. However, there are differences in the allelic frequency of certain *HLA-DRB1* SE alleles across different populations. For instance, HLA-DRB1*04:01 and HLA-DRB1*04:04 alleles are common in RA patients with Caucasian ancestry, while HLA-DRB1*04:05 allele is common in the Asian population [3, 6, 7, 9, 12–15]. It is also evident that the overall frequency of *HLA-DRB1* SE alleles in Asian populations is lower than in Caucasian populations, despite the similar RA prevalence between these populations. The reported population-specific risk allele HLA-DRB1*09:01 in the Japanese and Korean populations suggests genetic factors other than *HLA-DRB1* SE are associated with risk of RA development [3, 16].

For the past decade, the cost-effective computational approach to infer HLA alleles using single nucleotide polymorphisms (SNP) genotypes within the HLA region has become a preferable method to study the HLA region in large-scale genetic association studies. This approach has also enabled the integration of functional data from large genomic data to understand the pathogenesis of RA [17, 18]. For instance, the polymorphic amino acid residues at position 11 (e.g., valine, HLA-DRB1 Val11 or leucine, HLA-DRB1 Leu11) within HLA-DRB1 protein explained most of the genetic risk of developing ACPA-positive RA [17, 19–21], instead of the amino acid residues previously defined at positions 71 and 74, the conserved amino acid region of the SE alleles [2]. In addition, independent association signals between single amino acid position within other HLA proteins and risk of developing ACPA-positive RA demonstrated the importance of the HLA region in the pathogenesis of RA. For example, HLA-A amino acid residue at position 77 with asparagine residue (i.e., HLA-A Asn77), HLA-B amino acid residue at position 9 with aspartic acid (i.e., HLA-B Asp9), and HLA-DPB1 amino acid residue at position 9 with phenylalanine residue (i.e., HLA-DPB1 Phe9) are associated with risk of developing ACPA-positive RA [17, 21]. Furthermore, these predisposing HLA amino acid variants are located within the HLA molecules’ peptide-binding grooves, suggesting the role of antigen binding and involvement in antigen presentation in the adaptive immune response. A recent study of a Han Chinese population reported that the aspartic acid at position 160 within the HLA-DQA1 protein (i.e., HLA-DQA1 Asp160) was associated with an increased risk of developing ACPA-positive RA, instead of the well-described *HLA-DRB1* alleles [22]. Comparative modeling analysis showed that the additional negative charge of HLA-DQA1 Asp160 enhances the interaction between the dimers of the major histocompatibility complex (MHC) class II molecules, which may lead to an increase in T cell activation [22].

These findings were mainly reported in the Caucasian, African, and East Asian populations, and there is very limited information about RA-associated polymorphic HLA amino acid residues in Southeast Asian populations. There is a need to expand the field of study to multiple genetically dissimilar populations to investigate the implication of HLA risk factors in the disease pathogenesis.

Thus, we fine-mapped the *HLA* region in the ACPA-positive and ACPA-negative RA subsets from the multi-ethnic Malaysian population [23]. We further investigated the association between different HLA-DRB1 amino acid variants and compared the HLA-DRB1 amino acid haplotypes in different ethnic groups for risk of developing different subsets of RA.

## Methods

### Study design and study population

This study utilized data from the Malaysian Epidemiological Investigation of Rheumatoid Arthritis (MyEIRA), a large population-based case-control study of RA conducted in the multi-ethnic Malaysian population. The study design of MyEIRA has been described elsewhere [9, 24]. Briefly, this study analyzed data from 1260 patients with early RA (i.e., 530 Malays, 259 Chinese, 412 Indians, and 59 mixed ethnicities), and 1571 matched controls (i.e., 981 Malays, 206 Chinese, 297 Indians, and 87 with mixed ethnicities).

The RA cases were identified from nine rheumatology clinics throughout Peninsular Malaysia between 2005 and 2009. All RA cases were diagnosed according to the 1987 revised American College of Rheumatology (ACR) classification of rheumatoid arthritis criteria by rheumatologists. For each RA case, a control was randomly selected from the general population, matched for age, sex, and residential area. All study subjects were unrelated and ethnicity background was self-reported, based on questions about ancestry.

### Anti-citrullinated protein antibody measurement

The presence of ACPA in all individuals was assessed using anti-cyclic citrullinated peptide second-generation (anti-CCP2) ELISA kits (Immunoscan RA, Malmö, Sweden). Samples with results > 25 AU/mL were defined as ACPA-positive [9].

### HLA genotyping

The experimental classical *HLA* genotyping for *HLA-A*, *HLA-B*, *HLA-C*, *HLA-DRB1*, and *HLA-DQB1* genes was performed previously and described elsewhere [9, 25]. In brief, the *HLA* genotyping was performed for all DNA samples using the polymerase chain reaction and sequence-specific oligonucleotide probe hybridization (PCR-SSO) method (LABType® HLA test kits, One Lambda Inc., CA, USA) on the Luminex Multi-Analyte Profiling System (xMAP, Luminex Corporation, TX, USA). The *HLA* typing assignment was accomplished using the HLA Fusion software (version 1.3.0) provided by the manufacturer (One Lambda Inc., CA, USA).

### Dense SNP genotyping and quality controls

All individuals were genotyped using the Illumina iSelect HD custom genotyping array designed by the Immunochip Consortium (Immunochip, Illumina, Inc., San Diego, CA, USA). The Immunochip array was custom-designed with a dense coverage of HLA region to perform deep replication of major autoimmune and inflammatory diseases, including RA [23, 26]. The genotyping quality control (QC) was performed using PLINK v1.07 software [27]. The SNPs with call rate less than 99%, minor allele frequency (MAF) less than 0.01, and with significant departure from Hardy-Weinberg equilibrium (HWE) (*p*< 0.001), in both the RA cases and control groups, were excluded. Individuals with missing genotyping rate higher than 10% were also excluded. Then, a total of 25 individuals from the RA group (i.e., redundant RA and non-RA) were removed, followed by removal of a further 11 individuals (i.e., 6 RA cases and 5 matched normal controls) with missing genotyping rate > 10%, from the subsequent data analysis. A total of 113,576 SNPs in 2795 individuals (i.e., 1229 RA cases and 1566 controls) remained after QC. The individuals with mixed ethnicity parentage background were excluded from further analysis. Thus, the association testing was restricted to study subjects whose parents both came from the same ethnic group, giving a total of 1170 RA and 1479 controls for analysis after QC. The baseline demographic characteristics of the RA cases and controls are shown in Table 1.

**Table 1:** Demographic characteristics of patients with rheumatoid arthritis and controls in MyEIRA case-control study

Characteristics	All		Ethnicity					
			Malay		Chinese		Indian	
	RA cases* (*n* = 1229)	Controls* (*n* = 1566)	RA cases* (*n* = 514)	Controls* (*n* = 981)	RA cases* (*n* = 252)	Controls* (*n* = 204)	RA cases* (*n* = 404)	Controls* (*n* = 294)
Mean age (years old, SD)	48.11 ± 11.61	47.22 ± 11.37	46.18 ± 11.77	46.36 ± 11.40	52.27 ± 11.21	50.96 ± 11.31	47.92 ± 10.81	48.24 ± 10.58
Female (%)	85.8	85	86.2	85.7	80.9	82.8	87.3	82.3
ACPA positivity (%)	64.4	2.4	60.5	2.4	66.2	3.4	67.1	2.1
*HLA-DRB1* SE positivity (%)	40.1	16.2	35.6	12.9	36.5	12.3	48.3	29.9

All represent the total number of RA cases and matched controls for the Malay, Chinese, Indian and other/mixed ethnicities in this study. *MyEIRA* Malaysian Epidemiological Investigation of Rheumatoid Arthritis, *RA* rheumatoid arthritis, *SD* standard deviation, *ACPA* anti-citrullinated protein antibody, *HLA-DRB1 SE* HLA DR beta 1 shared epitope. *The number of individuals for the RA cases and controls was based on the number of individuals passing the dense SNP genotyping dataset quality control

### Imputation of classical HLA alleles and polymorphic amino acids residues

A total of 6152 SNPs between positions 29 and 34 Mb in the HLA region on chromosome 6 (GRCh37) were extracted from the post-QC Immunochip dataset. Using the extracted SNP genotypes from the *HLA* region, we imputed the HLA variants (i.e., classical 2-digit and 4-digit *HLA* alleles, and polymorphic amino acid residues of the *HLA* genes), along with the SNPs from the Pan-Asian reference panel [18, 19]. The Pan-Asian reference panel comprised 530 unrelated individuals of Asian descent: i.e., Han Chinese (*n* = 247, 46.6%), Malays (*n* = 120, 22.6%), Tamil Indians (*n* = 119, 22.4%), and Japanese (*n* = 44, 8.3%). The reference panel included a total of 6173 SNPs associated to 94 classical 2-digit *HLA* alleles, 179 classical 4-digit *HLA* alleles, and 1799 polymorphic amino acid positions [19, 28]. All the RA cases and controls were imputed together using the SNP2HLA software [18].

### HLA allele imputation accuracy assessment

We assessed the imputation accuracy for each imputed classical *HLA* allele in *HLA-A*, *HLA-B*, *HLA-C*, *HLA-DRB1*, and *HLA-DQB1* genes using experimental and imputed classical *HLA* genotype datasets from the normal controls with Malays, Chinese, and Indians. In brief, concordance rate was defined as the count of matched imputed classical *HLA* allele to the experimental classical *HLA* allele at the individual level, divided by the total count of observed experimental classical *HLA* allele within the studied population. Imputation accuracy assessment only considers individuals with available data for both experimental and imputed *HLA* genotypes. The *HLA* alleles’ distributions and their allelic frequencies vary in different populations/ethnic groups, so we further assessed the imputation accuracy in the three ethnic groups, i.e., Malays, Chinese, and Indians. Imputation accuracy with concordance rate above 90% was considered as high imputation accuracy threshold in this study.

### Association analysis of HLA alleles and amino acid polymorphisms

Referring to the data analysis described elsewhere, the logistic regression model was applied to test for the association between the imputed HLA variants and risk of developing different subsets of RA, separately in the Malay, Chinese, and Indian ethnic groups, with adjustment for age and sex [17, 19, 29]. The imputed HLA variants were defined by including the biallelic SNPs, classical 2-digit *HLA* alleles, classical 4-digit *HLA* alleles, and polymorphic HLA amino acid residues [17, 19, 29]. The analyses were conducted in PLINK v1.07 software [27]. The significance threshold of *p* value (*P*_*GWAS*_) was less than 5 × 10^−8^ in this study.

We implemented a stepwise logistic regression conditioned by the most associated variants, to search for the independent effects across the *HLA* region. All variables (i.e., imputed HLA variants) were systematically removed/added to obtain the best fit model based on the P_GWAS_ threshold. The Akaike information criterion (ΔAIC) and the improvement in the Bayesian information criterions (ΔBIC) were also considered to assess the best fit model. A modified version of a public Python 3.0 script (http://trevor-smith.github.io/stepwise-post/), which uses the Statsmodels module [30], was used in this analysis.

### HLA amino acid haplotype analysis

A group of RA-related classical *HLA-DRB1* alleles encoding a conserved amino acid sequence (^70^QRRAA^74^ or ^70^KRRAA^74^ or ^70^RRRAA^74^) at positions 70 to 74 in the third hypervariable region of the first domain of DRB1 was defined as shared epitope (SE) [2]. The *HLA-DRB1* SE alleles are the most established genetic risk factors for RA [2, 9, 31]. Nevertheless, the recent studies demonstrated that polymorphic HLA-DRB1 amino acid residues at positions 11 and 13 were the top association signals for risk of RA, instead of positions 70–74 [17, 19]. Hence, we aimed to replicate the investigation of HLA-DRB1 amino acid haplotypes and risk for ACPA-positive RA in the Caucasian and East Asian populations [17, 19], for the Malays, Chinese, and Indians.

We constructed the haplotypes manually based on the RA risk HLA-DRB1 haplotype model (i.e., defined by the polymorphic amino acid residues at positions 11, 13, 71, and 74), by filtering the subsets of HLA-DRB1 11-13-71-74 haplotypes in PLINK v1.07 software. First, we assessed the association between these HLA-DRB1 amino acid haplotypes and risk for ACPA-positive RA in all three ethnic groups. Then, we observed the risk effect (expressed as odds ratio, OR) between the published data and findings from the Malay, Chinese, and Indian ethnic groups.

### Meta-analysis and comparative analysis with published data

To test the generalizability of the polymorphic amino acid residues at position 11 within the HLA-DRB1 protein and risk of developing ACPA-positive RA in the Malay, Chinese, and Indian ethnic groups, we performed a meta-analysis using the Mantel-Haenszel method, with the random-effect model by means of cumulative OR with 95% confidence interval (95% CI). The heterogeneity between the studied ethnic groups was assessed using the Cochran Q-statistic (*P* < 0.10 considered significant). In addition, the *I*^*2*^ metric [*I*^*2*^ = (*Q* − df)/*Q*] was used to describe the percentage of variation across the different ethnic groups due to heterogeneity. *I*^*2*^ values of 25%, 50%, and 75% were considered as low, moderate, and high estimates, respectively. All analyses were performed in the PLINK v1.07 and Review Manager v5.3 (Copenhagen, The Nordic Cochrane Centre, The Cochrane Collaboration, 2014) software.

We compared the findings from this study with the published RA-associated genetic variants within the HLA region from different populations/ethnic groups (i.e., Caucasian, East Asian, African, and Han Chinese) to investigate the spectrum of association in the HLA region with risk of developing RA [17, 19–22]. Here, we restricted the selection of published RA-associated HLA variants to those computationally imputed from dense SNP genotypes within the HLA region.

## Results

### Imputed HLA variants and imputation accuracy assessment

We imputed a total of 3239 markers comprising 90 classical 2-digit *HLA* alleles, 175 classical 4-digit *HLA* alleles, 1799 specific HLA amino acid positions, and 1175 SNPs from the Pan-Asian reference panel. Our data demonstrated the overall concordance rate of the classical 2-digit HLA alleles satisfied the suggested concordance rate threshold of 90% for all five HLA genes, while decreased overall rates (ranged between 71.5 and 85.7%) were observed at 4-digit resolution (supplementary Table 1). Notably, the decreased overall concordance rates were attributed to the increased polymorphisms detected in these *HLA* genes. We further observed the concordance rates varied among the imputed classical *HLA* alleles at 2-digit and 4-digit resolutions for all *HLA* genes, where the variations were influenced by the distribution of the common/rare *HLA* alleles and its allelic frequency varies across different ethnic groups (supplementary Tables 2 and 3).

### HLA-DRB1 variants associated with risk of developing RA

The logistic regression analysis demonstrated that the genome-wide significant threshold (*P*_*GWAS*_) for association analysis was satisfied by 15 classical *HLA* class II alleles, which were located in *HLA-DRB1* (*n* = 6), *HLA-DQA1* (*n* = 4), and *HLA-DQB1* (*n* = 5) genes; 74 amino acid polymorphisms (47.3% in the HLA-DRB1 protein, 28.4% in the HLA-DQB1 protein, 20.3% in the HLA-DQA1 protein, and only 4.1% in the HLA Class I protein); and 128 SNP variants (supplementary Table 4). However, the distribution of these identified significant risk variants varied across the Malay, Chinese, and Indian ethnic groups, with the majority observed in the Malays.

In the Malay ethnic group, the most significant association across all variants tested was observed at HLA-DRB1 Val11 (OR = 4.26, 95% CI = 3.30–5.49, *P*_*GWAS*_ = 7.22 × 10^−29^), followed by HLA-DRB1 amino acid at position 120 with asparagine residue (i.e., HLA-DRB1 Asn120) (OR = 4.23, 95% CI = 3.28–5.45, *P*_*GWAS*_ = 8.59 × 10^−29^), which is in tight linkage disequilibrium (LD, D’ = 1.00) with HLA-DRB1 Val11 (Table 2 and supplementary Table 5)*.* Further peptide alignment analysis of the HLA-DRB1 protein revealed that the HLA-DRB1 Val11 and HLA-DRB1 Asn120 are exclusive characteristics for all the HLA-DRB1*04 and HLA-DRB1*10 alleles, indicating HLA-DRB1 Asn120 is not an independent risk factor for developing ACPA-positive RA among the Malay patients (online HLA alignment database https://www.ebi.ac.uk/cgi-bin/imgt/hla/align.cgi). Our observations implied that we have convincingly replicated the previous published data showing the association of the HLA-DRB1*04:05 allele (i.e., corresponding to HLA-DRB1 Val11 and HLA-DRB1 Asn120) with increased risk of ACPA-positive RA in the Malay ethnic group [9].

**Table 2:** Top association signals between HLA variants and risk of developing RA in the Malaysian population

Ethnicity	HLA variant	Position	Amino acid residue	Allele frequency		OR	95% CI	***P***
				RA cases	Controls			
**Overall RA**								
All	HLA-DRB1 position 120	32,657,518	Asparagine	0.25	0.12	2.46	2.13–2.84	**1.55 × 10**^− 34^*
Malay	HLA-DRB1 position 11	32,660,115	Valine	0.21	0.09	2.86	2.28–3.58	**4.11 × 10**^−20^*
Chinese	HLA-DRB1*04:05	32,660,042	–	0.13	0.04	3.47	2.00–6.03	9.49 × 10^−06^
Indian	HLA-DRB1 position 96	32,657,590	Histidine	0.42	0.53	0.64	0.51–0.79	6.16 × 10^−05^
**ACPA-positive RA**								
All	HLA-DRB1 position 120	32,657,518	Asparagine	0.31	0.12	3.34	2.84–3.92	**5.96 × 10**^−49^*
Malay	HLA-DRB1 position 11	32,660,115	Valine	0.28	0.09	4.26	3.30–5.49	**7.22 × 10**^−29^*
Chinese	HLA-DRB1*04:05	32,660,042	–	0.18	0.04	5.22	2.95–9.25	**1.52 × 10**^−08^*
Indian	HLA-DRB1 position 96	32,657,590	Histidine	0.37	0.53	0.48	0.37–0.62	**2.58 × 10**^− 08^*
**ACPA-negative RA**								
All	HLA-DRB1 position 60	32,659,968	Serine	0.15	0.12	0.63	0.54–0.75	7.09 × 10^−08^
Malay	HLA-DRB1 position 60	32,659,968	Serine	0.32	0.42	0.64	0.52–0.81	1.40 × 10^−04^
Chinese	HLA-B*27:04	31,431,272	–	0.06	0.02	4.40	1.55–12.53	5.51 × 10^−03^
Indian	HLA-B position 12	31,432,680	Valine	0.36	0.27	1.49	1.09–2.04	1.00 × 10^−02^

The table shows the top association signals between HLA variants and risk of developing ACPA-positive and ACPA-negative RA in the Malay, Chinese, and Indian ethnic groups. *All* combined group of individuals with Malay, Chinese, Indian, and others/mixed ethnicities, *RA* rheumatoid arthritis, *ACPA* anti-citrullinated protein antibody, *HLA* human leukocyte antigen, *DRB1* DR beta chain 1, *OR* odds ratio, *95% CI* 95% confidence interval, *P p* value. *Achieved genome-wide association threshold of *P*_*GWAS*_ < 5 × 10^−8^

Our findings in the Chinese ethnic group showed that HLA-DRB1*04:05 allele was the top association signal for risk of ACPA-positive RA (OR = 5.22, 95% CI = 2.95–9.25, *P*_*GWAS*_ = 1.52 × 10^−08^) (Table 2), in agreement with the previously published data using the experimental classical HLA genotype dataset [9]. Furthermore, we observed the association between HLA-DRB1 Asn120 and risk of ACPA-positive RA in the Chinese ethnic group; however, the signal was below the suggested *P*_*GWAS*_ threshold (OR = 3.05, 95% CI = 2.03–4.58, *P*_*GWAS*_ = 8.43 × 10^−08^). Moreover, HLA-DRB1*04:05 allele is one of the corresponding alleles to HLA-DRB1 Val11 and HLA-DRB1 Asn120, which are in tight LD (D’ = 1.00), based on the online database of HLA peptide sequence (supplementary Table 5, online HLA alignment database https://www.ebi.ac.uk/cgi-bin/ipd/imgt/hla/align.cgi). In view of the evidence for HLA-DRB1 Val11 as a common risk factor for ACPA-positive RA across different populations [17, 19, 20, 22], we tested the association between this variant and risk of ACPA-positive RA in the Chinese ethnic group. Our finding confirmed the increased risk of ACPA-positive RA in the Chinese ethnic group (OR = 2.87, 95% CI = 1.91–4.30, *P*_*GWAS*_ = 3.63 × 10^−7^), although this did not reach genome-wide significance (supplementary Table 5).

Further stratification analysis by ethnicity revealed the strongest association signal at amino acid position 96 within HLA-DRB1 peptide with histidine residue (i.e., HLA-DRB1 His96) among Indian patients with ACPA-positive RA (OR = 0.48, 95% CI = 0.37–0.62, *P*_*GWAS*_ = 2.58 × 10^−08^) (Table 2). The HLA-DRB1 peptide alignment showed that HLA-DRB1 His96 corresponded to specific alleles from HLA-DRB1*03/*07/*08/*09/*11/*12/*13/*14 allele groups (online HLA alignment database https://www.ebi.ac.uk/cgi-bin/ipd/imgt/hla/align.cgi). Of these *HLA-DRB1* alleles, the HLA-DRB1*13 allele group was inversely associated with the risk of ACPA-positive RA in the Caucasian, Japanese, and Indian Tamil populations in previous studies [32–34]. Although the commonly shared variant of HLA-DRB1 Val11 also increased the risk of ACPA-positive RA among the Indian patients, this association did not reach the genome-wide significant threshold (OR = 1.99, 95% CI = 1.52–2.61, *P*_*GWAS*_ = 6.60 × 10^−07^) (supplementary Table 5).

We did not observe any significant association between the imputed HLA variants and risk of developing ACPA-negative RA in any of the three major ethnic groups (data not shown).

### Risk factor independent from HLA-DRB1 in the ACPA-positive RA subset

**Fig. 1:**
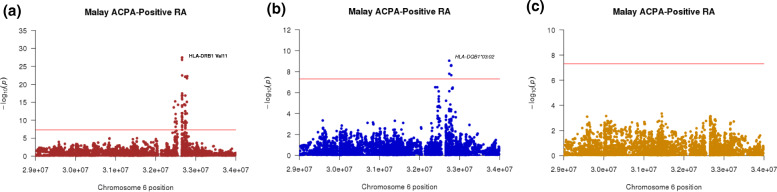
Association plots of the tested variants in the HLA region to ACPA-positive RA in Malays. Legends: **a** Regional plot of stepwise logistic regression in ACPA-positive Malay RA patients, HLA-DRB1 Val11 mapped as the strongest association signal (*P*_GWAS_ = 2.4 × 10^−35^). **b** Conditioning of HLA-DRB1 Val11, HLA-DQB1*03:02 alleles (*P*_GWAS_ = 1.57 × 10^−9^) were mapped as the second independent HLA risk variants with decreased risk of developing ACPA-positive RA. **c** No independent variants observed from conditioning the two most associated HLA variants, i.e., HLA-DRB1 Val11 and HLA-DQB1*03:02 allele. The red line represents the suggested significant threshold, i.e., genome-wide significant threshold of *P*_*GWAS*_ < 5 × 10^−8^

To look for independent effects across the HLA region, we conducted a stepwise logistic regression. Conditioning by the most associated risk variant, i.e., HLA-DRB1 Val11, with ACPA-positive RA in the Malay ethnic group revealed an inverse association of HLA-DQB1*03:02 allele with risk of developing ACPA-positive RA (OR = 0.17, 95% CI = 0.09–0.30, *P*_*GWAS*_ = 1.60 × 10^−09^) (Fig. 1). No further independent risk variants were detected within the HLA region for ACPA-positive RA in the Malay ethnic group. This finding was confirmed by using the experimental classical *HLA* genotype dataset that demonstrated an inverse association between HLA-DQB1*03:02 allele and risk of ACPA-positive RA (OR = 0.27, 95% CI = 0.17–0.45, *p* = 2.30 × 10^−07^) (supplementary Table 6).

“The stepwise logistic regression demonstrated that the top association signals for the Chinese and Indian ethnic groups were HLA-DRB1*04:05 allele (OR = 3.99, 95% CI = 2.22-7.18, *p* = 3.90 × 10^−06^) and HLA-DRB1 His96 (OR = 0.40, 95% CI = 0.30–0.52, *p* = 5.25 × 10^−08^), respectively. However, these two variants did not satisfy the genome-wide significant threshold (supplementary figure 1). Conditioning on these top association signals showed that no further independent HLA risk variants were detected for ACPA-positive RA in the Chinese and Indian ethnic groups.”

### Comparative analysis for the independent effects of HLA amino acid variants and risk of ACPA-positive RA across different populations

We compared the published independent RA-associated polymorphic HLA amino acid positions across different populations/ethnic groups and the data is presented in Table 3. The HLA-DRB1 Val11 was the most common HLA amino acid variant significantly associated (*P*_*GWAS*_< 5 × 10^−08^) with increased risk of ACPA-positive RA in all the studied populations included in this study (Table 3), and this association was validated in our study among the Malay ethnic group. Within the same HLA protein, amino acid position 13 with histidine residue was associated with increased risk for ACPA-positive RA in the East Asian and African populations; it was, however, in tight LD with HLA-DRB1 Val11. Furthermore, different amino acid positions, i.e., positions 37, 57, and 74, were reported as RA-associated genetic variants in the ACPA-positive RA from the East Asian population (Table 3). We did not observe the RA-associated polymorphic HLA-DRB1 amino acid at positions 13, 37, 57, and 74 associated with the risk of developing ACPA-positive RA in our study population. However, the independent effect of HLA-DRB1 His96 associated with decreased risk for ACPA-positive RA in the Malaysian Indian patients was not reported in any of the published data from these studied populations.

**Table 3:** Independent specific amino acid residues associated with risk of developing ACPA-positive RA in different populations

Region	Population	RA cases	Controls	HLA locus	Amino acid position	Residue	OR	***P***	***χ***^**2**^	***P***_**omnibus**_	Ref
Europe	Caucasian	5018	14,974	HLA-DRB1	11	Valine	3.80	–	2260.0	1.0 × 10^−581^	[17]
				HLA-B	9	Aspartic acid	2.12	–	166.0	2.0 × 10^−37^	
				HLA-DPB1	9	Phenylalanine	1.40	–	93.3	1.0 × 10^−20^	
	Caucasian	7279	15,870	HLA-DRB1	11	Valine	–	–	3551.5	1.0 × 10^−692^	[21]
				HLA-B	9	Aspartic acid	1.93	1.0 × 10^−36^	160.2	–	
				HLA-DPB1	9	Phenylalanine	1.31	1.0 × 10^−19^	82.6	–	
				HLA-A	77	Asparagine	0.85	2.7 × 10^−08^	30.9	–	
East Asia	Korean	616	675	HLA-DRB1	11	–	–	–	–	6.1 × 10^−36^	[17]
				HLA-DRB1	13	–	–	–	–	3.1 × 10^−36^	
	Korean and Chinese	2782	4315	HLA-DRB1	13	Histidine	2.03	–	–	6.9 × 10^−135^	[19]
				HLA-DRB1	57	–	–	–	–	2.2 × 10^−33^	
				HLA-DRB1	74	–	–	–	–	1.1 × 10^−08^	
				HLA-DRB1	11	–	–	–	–	1.7 × 10^−129^	
				HLA-B	9	Aspartic acid	4.21	3.8 × 10^−06^	21.4	–	
				HLA-DPB1	9	Phenylalanine	1.26	3.0 × 10^−05^	17.4	–	
	Han Chinese	961	1812	HLA-DQA1	160	Aspartic acid	2.29	6.2 × 10^−36^	156.6	–	[22]
				HLA-DRB1	11	Valine	1.79	2.1 × 10^−13^	53.9	–	
				HLA-DRB1	37	Asparagine	0.49	5.8 × 10^−16^	65.5	–	
Africa	African	266	362	HLA-DRB1	11	Valine	5.1	3.4 × 10^−26^	112.1	–	[20]
				HLA-DRB1	13	Histidine	6.1	1.2 × 10^−27^	118.7	–	
Malaysian*	Malay	311	981	HLA-DRB1	11	Valine	4.26	7.2 × 10^−29^	121.1	–	–
	Chinese	167	204	HLA-DRB1	11	Valine	2.87	3.6 × 10^−07^	25.9	–	–
	Indian	195	294	HLA-DRB1	96	Histidine	0.48	2.2 × 10^−08^	31.3	–	–

The table compares the reported independent association between HLA amino acid variants and the risk of developing ACPA-positive RA in different populations. *ACPA* anti-citrullinated protein antibody, *HLA* human leukocyte antigen, *DRB1* DR beta 1, *DPB1* DP beta 1, *DQA1* DQ alpha 1, *DQB1* DQ beta 1, *OR* odds ratio, *P p* value; *χ*^2^ chi-square, *P*_*omnibus*_ omnibus *p* value. *The Malaysian population comprising different ethnic groups: the Malay (i.e., predominant southeast Asian ethnic group) [25], Chinese (i.e., Han Chinese descendants of immigrants who arrived during the nineteenth and early twentieth from Southern China) [35], and Indian (Tamil descendants of immigrants who arrived during the nineteenth and early twentieth century from Southern India) [36]

This observation supported the genetic association of the HLA region to RA and that this is commonly attributed to *HLA-DRB1* genes. Furthermore, the observed risk effects of the different amino acids from the same HLA-DRB1 protein suggested that while some may promote the pathogenic process in RA, others may counteract the process.

We further observed that the polymorphic HLA amino acid positions independent of *HLA-DRB1* gene were associated with the risk of developing ACPA-positive RA in a population-specific manner. For instance, HLA-A Asn77, HLA-B Asp9, and HLA-DPB1 Phe9 were reported as RA-associated genetic variants in the Caucasian populations, while HLA-DRB1 His13 was RA-associated in the East Asian and African populations. More recently, the HLA-DQA1 Asp160 was reported in Han Chinese to be associated with an increased risk of ACPA-positive RA. However, we did not observe any association for these amino acid variants with ACPA-positive RA in our study population with Malay, Chinese, or Indian origins.

To summarize, these predisposing HLA-specific amino acid positions may exhibit shared-genetic component or population-specific risk signals, suggesting the existence of ethnogenetic heterogeneity in the RA population.

### HLA-DRB1 amino acid haplotypes as risk factors for ACPA-positive RA

Of the 16 possible HLA-DRB1 amino acid haplotypes at positions 11, 13, 71, and 74 [17], we observed only 10, 12, and 12 haplotypes, respectively, in Malay, Chinese, and Indian ethnic groups to be associated with ACPA-positive RA (Table 4). Our findings revealed that the Val^11^-His^13^-Arg^71^-Ala^74^ haplotype was strongly associated with risk of ACPA-positive RA in the Malay (OR = 5.28, 95% CI = 3.06–9.09, *p* = 1.22 × 10^−09^), Chinese (OR = 10.33, 95% CI = 4.39–24.31, *p* = 9.81 × 10^−09^), and Indian (OR = 3.84, 95% CI = 3.75–4.75, *p* = 0.03) populations (Table 4). Meanwhile, we observed the Val^11^-Phe^13^-Arg^71^-Ala^74^ haplotype was associated with increased risk of ACPA-positive RA in the Malays (OR = 4.35, 95% CI = 2.27–8.32, *p* = 9.78 × 10^−06^) and Indians (OR = 2.00, 95% CI = 1.10–3.68, *p* = 0.03), but not in the Chinese. Interestingly, while Ser^11^-Ser^13^-Glx^71^-Ala^74^ conferred significant risk for ACPA-positive RA among the Chinese (OR = 12.91, 95% CI = 2.55–65.34, *p* = 6.98 × 10^−04^), it demonstrated an inverse association to ACPA-positive RA in the Indian population (OR = 0.40, 95% CI = 0.18–0.86, *p* = 0.03).

**Table 4:** HLA-DRB1 amino acid haplotypes and risk of ACPA-positive RA in different populations

HLA-DRB1 amino acid at position				Malay^**#**^		Chinese^**#**^		Indian^**#**^		European^**a**^		European^**b**^		East Asian^**c**^		Classical ***HLA-DRB1*** alleles
11	13	71	74	OR	95% CI	OR	95% CI	OR	95% CI	OR	95% CI	OR	95% CI	OR	95% CI	
Pro	Arg	Ala	Ala	***Ref***		***Ref***	–	***Ref***		***Ref***		***Ref***		***Ref***		*15:01, *15:02, *15:04, *16:02
Val	His	Arg	Ala	5.28	3.06–9.09	10.33	4.39–24.31	3.84	1.02–14.44	4.22	3.75–4.75	3.63	3.29–4.01	3.02	2.62–3.48	*04:04, *04:05, *04:10
Val	Phe	Arg	Ala	4.35	2.27–8.32	–	–	2.00	1.10–3.68			4.65	3.80–5.70	2.83	2.22–3.61	*10:01
Ser	Ser	Glx	Ala	0.36	0.11–1.22	12.91	2.55–65.34	0.40	0.18–0.86	0.59	0.51–0.68	0.60	0.54–0.67	0.6	0.50–0.72	*13:01, *13:02
Ser	Ser	Lys	Arg	0.64	0.30–1.37	0.81	0.27–2.40	0.82	0.37–1.78	0.63	0.54–0.73	0.67	0.60–0.76	0.71	0.53–0.96	*03:01
Gly	Tyr	Arg	Gln	0.78	0.46–1.34	1.61	0.28–9.41	0.74	0.42–1.30	0.91	0.80–1.03	0.92	0.83–1.02	0.9	0.75–1.08	*07:01
Ser	Gly	Arg	Ala	0.83	0.57–1.22	1.67	0.76–3.66	1.28	0.50–3.30	0.88	0.77–1.00	1.04	0.86–1.25	1.12	0.95–1.32	*12:01, *12:02, *12:03
Ser	Ser	Arg	Ala	0.72	0.29–1.81	2.69	1.02–7.07	0.56	0.22–1.41			0.76	0.67–0.86	0.83	0.68–1.02	*1101, *11:05, *13:12
Val	His	Arg	Glx	0.72	0.29–1.81	1.88	0.66–5.38	1.09	0.57–2.07	1.65	1.24–2.19	1.29	1.06–1.57	0.95	0.79–1.13	*0403, *04:06
Ser	Gly	Arg	Glx	1.08	0.49–2.39	1.21	0.30–4.96	0.72	0.38–1.37	–	–	0.49	0.26–0.91	0.56	0.42–0.74	*14:04
Ser	Gly	Arg	Leu	–	–	1.41	0.51–3.87	0.87	0.25–3.00	0.71	0.57–0.89	0.83	0.70–0.98	0.85	0.72–1.01	*08:01, *08:02, *08:03, *08:09
Ser	Ser	Arg	Glx	–	–	1.34	0.43–4.24	–	–	0.84	0.67–1.05	0.77	0.64–0.94	0.77	0.60–0.99	*14:01, *14:05,*14:07
Ser	Ser	Arg	Leu	–	–	1.41	0.51–3.87	–	–	–	–	–	–	–	–	*14:03
Val	His	Lys	Ala	–	–	–	–	1.92	0.67–5.51	4.44	4.02–4.91	4.03	3.72–4.37	3.63	2.63–5.00	*04:01
Leu	Phe	Arg	Ala	–	–	–	–	–	–	2.17	1.94–2.42	2.11	1.94–2.31	1.51	1.26–1.80	*01:01, *01:02
Asp	Phe	Arg	Glx	–	–	–	–	–	–	1.65	1.29–2.10	1.82	1.52–2.18	1.8	1.56–2.09	*09:01
Pro	Arg	Arg	Ala	–	–	–	–	–	–	2.04	1.59–2.62	1.58	1.26–1.99	1.21	0.85–1.73	*16:01, *16:02
Val	His	Glx	Ala	–	–	–	–	–	–	1.43	1.04–1.96	1.03	0.71–1.50	–	–	*04:02, *04:37
Ser	Ser	Lys	Ala	–	–	–	–	–	–	1.04	0.76–1.41	0.87	0.66–1.14	–	–	*13:03
Leu	Phe	Glx	Ala	–	–	–	–	–	–	0.73	0.42–1.27	0.71	0.55–0.93	–	–	*01:03

*ACPA* anti-citrullinated protein antibody, *HLA* human leukocyte antigen, *DRB1* DR beta chain 1, *OR* odds ratio, *95% CI* 95% confidence interval. Amino acid abbreviations: *Pro* proline, *Arg* arginine, *Ala* alanine, *Val* valine, *His* histidine, *Phe* phenylalanine, *Ser* serine, *Glx* glutamic acid/glutamine, *Gly* glycine, *Tyr* tyrosine, *Gln* glutamine, *Leu* leucine, *Lys* lysine. ^#^The individuals with Malay, Chinese, and Indian ethnicity recruited for Malaysian Epidemiological Investigation of Rheumatoid Arthritis (MyEIRA) population-based case-control study. ^a^Published data retrieved from Raychaudhuri et al. [17]. ^b^Published data retrieved from Han et al. (2014) [21]. ^c^Published data retrieved from Okada et al. (2014) [37]

Comparing our findings from the multi-ethnic Malaysian population with the published data from other populations with European and East Asian origins, the Val11-His13-Arg71-Ala74 was the most significant and commonly shared risk factor among the European and Asian populations (Table 4). The decreased risk of ACPA-positive RA associated with the Ser^11^-Ser^13^-Glx^71^-Ala^74^ haplotype observed in the European and East Asian populations was consistently replicated in the Malaysian Indian ethnic group. In contrast, this haplotype conferred risk for ACPA-positive RA in the Malaysian Chinese ethnic group. Notably, this haplotype is encoded by HLA-DRB1*13:01 and HLA-DRB1*13:03 alleles. It has been previously reported that the HLA-DRB1*13 allele has a dual role: as genetic modulator of ACPA positivity, whereby it was inversely associated with risk of ACPA-positive RA; but also, in combination with HLA-DRB1*03, it decreased the risk of ACPA-negative RA [36]. Our observation in the Malaysian Chinese ethnic group was not in line with the inverse association to ACPA-positive reported in the Caucasian and East Asian populations, suggesting different immune reactions may occur in RA with different ethnicity/population backgrounds.

### Amino acid polymorphisms at position 11 within HLA-DRB1 protein and risk of RA

**Fig. 2:**
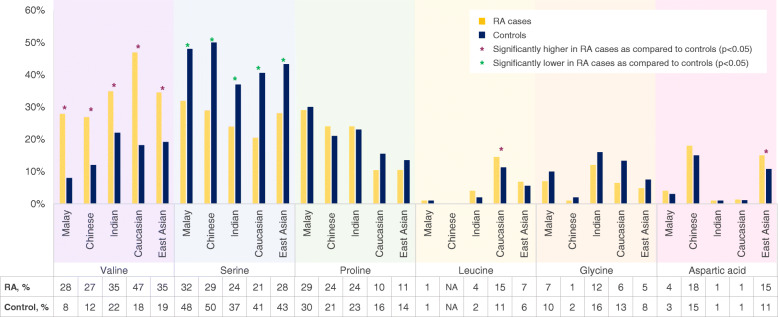
The frequency spectra of amino acid variants at position 11 within HLA-DRB1 protein. Legends: The figure illustrates the frequency of amino acid residues at position 11 within the HLA-DRB1 protein for the Malay, Chinese, and Indian ethnic groups. The asterisk indicates the published data retrieved from Okada et al. (2014) [19], and the number sign indicates the published data retrieved from Raychaudhuri et al. [17]

We investigated the frequency of the polymorphic amino acid residues (i.e., valine, serine, proline, leucine, glycine, and aspartic acid) at position 11 in the HLA-DRB1 protein of the Malaysian population with Malay, Chinese, and Indian origins and further compared these frequencies with the published data from Caucasian and East Asian populations [19]. Our data demonstrated that while the frequency of valine residue was higher in RA cases as compared to the normal control group in all the populations, the frequency of serine residue was lower in the RA cases in comparison with the normal controls (Fig. 2). Interestingly, leucine residue, which encodes the classical HLA-DRB1*01 alleles, was commonly found among the individuals of European ancestry (> 10% in both RA cases and control group), but was found in less than 5% of the Malay and Indian ethnic groups, and was absent in the Chinese RA cases and controls. It is noteworthy that the aspartic acid residue was commonly found in the Chinese individuals. This amino acid residue corresponds to the classical HLA-DRB1*09, an allele which was reported as a risk factor for RA development, independent of the *HLA-DRB1 SE* alleles [19]. The frequencies of proline and glycine residues, which encode the classical HLA-DRB1*15 and HLA-DRB1*07 alleles respectively, were comparable between RA cases and control group for all populations (Fig. 2).

Next, we performed meta-analyses to investigate the generalizability of the effect of the polymorphic HLA-DRB1 amino acid residues at position 11 on the risk for ACPA-positive RA in the Malay, Chinese, and Indian ethnic groups. Our finding demonstrated significant cumulative OR of the HLA-DRB1 Val11 for risk of ACPA-positive RA (OR_cumulative_ 2.86 2.90, *p* < 0.0001); however, we observed high heterogeneity within studies (*I*^*2*^ =91%) (supplementary Figure 2a). Interestingly, we observed a decreased risk of developing ACPA-positive RA associated with serine and glycine residues (i.e., serine, OR_cumulative_ 0.49, *p* < 0.00001, *I*^*2*^ =0%; glycine, OR_cumulative_ 0.68, *p* < 0.002, *I*^*2=*^ 0%) (Supplementary Figure. 2b and c).

## Discussion

Our study confirmed that the HLA-DRB1 genes with their functional characteristics are the major determinants in the pathogenesis of RA, specifically in the ACPA-positive RA subset in the multi-ethnic Malaysian population, supporting the notion of shared RA risk across different populations. We found HLA-DRB1 Val11 conferred the strongest risk effect in the ACPA-positive RA in the Malay population, one of the predominant ethnic groups in Southeast Asia. Additionally, HLA-DQB1*03:02 demonstrated a novel and independent protective effect for ACPA-positive RA in the Malay group. Interestingly, Indian RA patients carrying HLA-DRB1 His96 are protected from risk of developing ACPA-positive RA.

The observed RA risk of HLA-DRB1 Val11 in our study population is generally concordant with the published data from different large-scale genetic association studies of Caucasian, African, and East Asian populations, in terms of the amino acid position as well as magnitude of risk. It is notable that HLA-DRB1 Val11 is located within the peptide-binding groove of the HLA Class II molecules. This suggests the pathogenic role of the identified amino acid at position 11 of the HLA-DRB1 protein (i.e., HLA-DRB1 position 11), which enables peptide binding and further recognition of MHC-peptide complexes by T cells involved in providing help to B cells expressing and producing ACPA IgG. Future study of this replicated and validated risk variant, i.e., HLA-DRB1 position 11, is needed to generate new insights and better understanding of the implication of the risk variant for the pathophysiology of ACPA-positive RA.

The valine or leucine residue at position 11 within the HLA-DRB1 protein (i.e., HLA-DRB1 Val11 and HLA-DRB1 Leu11) is associated, predominately in the Caucasian and Spanish populations, with increased risk of severe radiographic progression in ACPA-positive RA, independent of *HLA-DRB1 SE* status [38]. The present extension of this observation to other populations, including the Malaysian population, may lead to better understanding of the pathogenic role of HLA-DRB1 Val11 and/or HLA-DRB1 Leu11 and their effect on the clinical phenotype of the disease. In this current study, the clinical data from the recruited RA cases were limited. Future studies of the implications of the identified RA risk factor on the disease progression will provide new insights/knowledge that may aid in the characterization of the RA phenotype in the clinical setting.

HLA-DRB1 His13 was reported to have the strongest association with risk of ACPA-positive RA in a mixed East Asian population comprising South Korean and Han Chinese in Beijing [19], while an earlier study in a homogenous Korean population demonstrated HLA-DRB1 Val11 was strongly associated with risk for ACPA-positive RA [17]. However, both HLA-DRB1 Val11 and HLA-DRB1 His13 are in tight LD. The HLA-DRB1 His13 observed in the mixed population study could be due to the influence of the different genetic profile of Han Chinese individuals (16.8% in RA cases and 20.2% in controls). Although the South Korean and Chinese populations have common ancestry, the genetic profiles of these populations are distinctive [39].

Interestingly, our findings demonstrated HLA-DQB1*03:02 allele as a novel potentially protective factor regarding risk of developing ACPA-positive RA in the Malay ethnic group. Of a different note, the HLA-DQB1*03:02 allele was reported to associate with increased risk of developing celiac disease in the Iranian population [40]. Taken together, it is suggested that HLA-DQB1*03:02 allele may have opposing effects, being a protective allele in one disease and a risk factor in another disease.

Recently, aspartic acid residue at position 160 within the HLA-DQA1 protein was reported to be the most significant risk factor for ACPA-positive RA in the Han Chinese population of Beijing, with HLA-DRB1 Val11 as the second strongest risk factor [22]. This pattern was however not observed in the Malaysian Chinese ethnic group in our study. The most plausible explanation is the genetic differences between the Beijing Han Chinese and the Malaysian Chinese. The Malaysian Chinese are mainly descendants of nineteenth and early twentieth century Han Chinese immigrants from Southern China (particularly the provinces of Fujian, Guandong, and Hainan) [35]. Furthermore, genetic population studies have shown that the Southern and Northern Han Chinese are two distinctive populations [39, 41].

High imputation accuracy observed in our studied dataset suggested the suitability of the Caucasian-based Immunochip microarray [23] and usefulness of the admixture Pan-Asian reference panel [19] for HLA imputation in the multi-ethnic Malaysian population. Based on these local evidences, utilizing the Immunochip microarray and admixture Pan-Asian reference panel for fine-mapping of HLA variants in other autoimmune diseases such as systemic lupus erythematosus, multiple sclerosis, and ankylosing spondylitis can be recommended.

## Conclusions

Our new findings in Southeast Asian populations are in concordance with the data from other populations, suggesting HLA-DRB1 Val11 valine as the most important genetic component for the risk of ACPA-positive RA. Notably, our data also showed a novel protective allele in the *HLA-DQB1* gene (i.e., HLA-DQB1*03:02) associated with the risk of developing ACPA-positive RA in the Malay ethnic group. The different risk and protective residues of HLA-DRB1 amino acid at positions 11 and 96 in the Malay and Indian patients with ACPA-positive suggested different amino acid residues within the same HLA protein may promote or counteract the pathogenesis of RA. In common with the Caucasian population, there is little risk from HLA locus for ACPA-negative RA in the multi-ethnic Malaysian population.

## Supplementary Information

**Additional file 1: Supplementary Table 1.** Overall concordance rate of *HLA-A*, *HLA-B, HLA-C, HLA-DRB1* and *HLA-DQB1* genes in the control group, stratified by ethnicity. **Supplementary Table 2.** Classical 2-digit HLA alleles imputation accuracy in the control group, stratified by ethnicity. **Supplementary Table 3.** Classical 4-digit HLA alleles imputation accuracy in the control group, stratified by ethnicity. **Supplementary Table 4.** Number of imputed classical HLA alleles and amino acid polymorphisms achieved published genome-wide threshold of *p*< 5 × 10^− 08^. **Supplementary Table 5.** Logistic regression results of the association between imputed HLA amino acids and alleles, and risk of developing ACPA-positive rheumatoid arthritis in the Malay, Chinese and Indian ethnic groups. **Supplementary Table 6.** Stepwise logistic regression analysis for risk of ACPA-positive RA in the Malay ethnic group. **Supplementary Figure 1.** Plot of stepwise logistic regression analysis to fine-map HLA variants as risk factor for ACPA-positive RA in the Chinese and Indian ethnic groups. **Supplementary Figure 2.** Meta-analysis of polymorphic HLA-DRB1 amino acid residues position 11 and risk of developing ACPA-positive RA in Malay, Chinese and Indian ethnic groups.

## Data Availability

All data generated or analyzed during this study are included in this published article and its supplementary information files.

## Abbreviations

HLA: Human leukocyte antigen
RA: Rheumatoid arthritis
HLA-A: Human leukocyte antigen A
HLA-B: Human leukocyte antigen B
HLA-DPB1: Human leukocyte antigen DP beta 1
HLA-DQA1: Human leukocyte antigen DQ alpha 1
ACPA: Anti-citrullinated protein antibody
MyEIRA: Malaysian Epidemiological Investigation of Rheumatoid Arthritis
OR: Odds ratio
95% CI: 95% confidence interval
P_GWAS_: Genome-wide significance threshold of less than 5 × 10^−08^
*HLA-DRB1* SE: Human leukocyte antigen DR beta 1 shared epitope
SNP: Single nucleotide polymorphisms
HLA-DRB1 Val11: HLA-DRB1 amino acid at position 11 with valine residue
HLA-DRB1 Leu11: HLA-DRB1 amino acid at position 11 with leucine residue
HLA-A Asn77: HLA-A amino acid at position 77 with asparagine residue
HLA-B Asp9: HLA-B amino acid at position 9 with aspartic acid
HLA-DPB1 Phe9: HLA-DPB1 amino acid at position 9 with phenylalanine residue
HLA-DQA1 Asp160: HLA-DQA1 amino acid at position 160 with aspartic acid
MHC: Major histocompatibility complex
ACR: American College of Rheumatology
anti-CCP2: Anti-cyclic citrullinated peptide second-generation (anti-CCP2)
PCR-SSO: Polymerase chain reaction sequence-specific oligonucleotide
QC: Quality control
MAF: Minor allele frequency
HWE: Hardy-Weinberg equilibrium
AIC: Akaike information criterion
BIC: Bayesian information criterion
HLA-DRB1 Asn120: HLA-DRB1 amino acid at position 120 with asparagine residue
LD: Linkage disequilibrium
HLA-DRB1 His96: HLA-DRB1 amino acid at position 96 with histidine residue
Pro: Proline
Arg: Arginine
Ala: Alanine
Val: Valine
His: Histidine
Phe: Phenylalanine
Ser: Serine
Glx: Glutamic acid/Glutamine
Gly: Glycine
Tyr: Tyrosine
Gln: Glutamine
Leu: Leucine
Lys: Lysine
