# Supplementary material for: The spectrum of association in HLA region with rheumatoid arthritis in a diverse Asian population: evidence from the MyEIRA case-control study
Source: Arthritis Res Ther. 2021 Jan 30;23:46. doi: 10.1186/s13075-021-02431-z (PMC7847037; doi:10.1186/s13075-021-02431-z)
Supplement: Supplementary file 1 — Additional file 1: Supplementary Table 1. Overall concordance rate of HLA-A, HLA-B, HLA-C, HLA-DRB1 and HLA-DQB1 genes in the control group, stratified by ethnicity. Supplementary Table 2. Classical 2-digit HLA alleles imputation accuracy in the control group, stratified by ethnicity. Supplementary Table 3. Classical 4-digit HLA alleles imputation accuracy in the control group, stratified by ethnicity. Supplementary Table 4. Number of imputed classical HLA alleles and amino acid polymorphisms achieved published genome-wide threshold of p< 5 × 10− 08. Supplementary Table 5. Logistic regression results of the association between imputed HLA amino acids and alleles, and risk of developing ACPA-positive rheumatoid arthritis in the Malay, Chinese and Indian ethnic groups. Supplementary Table 6. Stepwise logistic regression analysis for risk of ACPA-positive RA in the Malay ethnic group. Supplementary Figure 1. Plot of stepwise logistic regression analysis to fine-map HLA variants as risk factor for ACPA-positive RA in the Chinese and Indian ethnic groups. Supplementary Figure 2. Meta-analysis of polymorphic HLA-DRB1 amino acid residues position 11 and risk of developing ACPA-positive RA in Malay, Chinese and Indian ethnic groups. [file 13075_2021_2431_MOESM1_ESM.docx]

**Supplementary table 1** Overall concordance rate of *HLA-A*, *HLA-B, HLA-C, HLA-DRB1* and *HLA-DQB1* genes in the control group, stratified by ethnicity

|  | **Overall concordance rate (%)** | | |
| --- | --- | --- | --- |
|  | **Classical 2-digit HLA alleles** |  | **Classical 4-digit HLA alleles** |
| **All** |  |  |  |
| *HLA-A* | 96.3 |  | 70.1 |
| *HLA-B* | 91.0 |  | 72.7 |
| *HLA-C* | 94.5 |  | 85.7 |
| *HLA-DRB1* | 91.3 |  | 71.5 |
| *HLA-DQB1* | 91.7 |  | 85.6 |
| **Malay** |  |  |  |
| *HLA-A* | 95.9 |  | 70.8 |
| *HLA-B* | 91.7 |  | 64.9 |
| *HLA-C* | 94.6 |  | 88.2 |
| *HLA-DRB1* | 90.5 |  | 69.3 |
| *HLA-DQB1* | 90.2 |  | 81.4 |
| **Chinese** |  |  |  |
| *HLA-A* | 98.3 |  | 68.1 |
| *HLA-B* | 89.8 |  | 75.3 |
| *HLA-C* | 97.3 |  | 85.0 |
| *HLA-DRB1* | 97.5 |  | 76.5 |
| *HLA-DQB1* | 96.6 |  | 92.2 |
| **Indian** |  |  |  |
| *HLA-A* | 96.2 |  | 76.7 |
| *HLA-B* | 90.2 |  | 72.2 |
| *HLA-C* | 92.7 |  | 87.1 |
| *HLA-DRB1* | 94.2 |  | 79.2 |
| *HLA-DQB1* | 92.8 |  | 85.5 |

All: combined group of individuals with Malays, Chinese, Indians and other/mixed ethnicities. Concordance rate (%) is the total number of imputed classical HLA alleles concordant with experimental classical HLA alleles divided by the total number of detected experimental classical HLA alleles within the population.

**Supplementary table 2** Classical 2-digit HLA alleles imputation accuracy in the control group, stratified by ethnicity

| **HLA genes** | **Ethnicity** | ***HLA* alleles**^a^ | **gHLA allele count** | **Matched iHLA allele count** | **Concordance rate (%)** |
| --- | --- | --- | --- | --- | --- |
| ***HLA-A*** | **All^#^** | *24 | 1602 | 1549 | 96.69 |
|  |  | *11 | 1186 | 1131 | 95.36 |
|  |  | *02 | 1119 | 1051 | 93.92 |
|  |  | *33 | 584 | 568 | 97.26 |
|  |  | *01 | 302 | 292 | 96.69 |
|  |  | *34 | 204 | 186 | 91.18 |
|  |  | *03 | 157 | 151 | 96.18 |
|  |  | *26 | 117 | 111 | 94.87 |
|  |  | *68 | 109 | 102 | 93.58 |
|  |  | *31 | 71 | 65 | 91.55 |
|  |  | *32 | 60 | 55 | 91.67 |
|  |  | *30 | 45 | 39 | 86.67 |
|  |  | *29 | 43 | 41 | 95.35 |
|  | **Malay^#^** | *24 | 739 | 721 | 97.56 |
|  |  | *11 | 356 | 343 | 96.35 |
|  |  | *02 | 315 | 304 | 96.51 |
|  |  | *33 | 248 | 245 | 98.79 |
|  |  | *34 | 117 | 111 | 94.87 |
|  |  | *01 | 48 | 45 | 93.75 |
|  |  | *03 | 25 | 23 | 92.00 |
|  |  | *26 | 31 | 29 | 93.55 |
|  |  | *68 | 23 | 23 | 100.00 |
|  |  | *29 | 8 | 8 | 100.00 |
|  |  | *32 | 10 | 10 | 100.00 |
|  |  | *30 | 14 | 13 | 92.86 |
|  |  | *31 | 8 | 7 | 87.50 |
|  | **Chinese^#^** | *11 | 137 | 135 | 98.54 |
|  |  | *02 | 125 | 122 | 97.60 |
|  |  | *24 | 68 | 65 | 95.59 |
|  |  | *33 | 51 | 51 | 100.00 |
|  |  | *26 | 13 | 13 | 100.00 |
|  |  | *31 | 6 | 6 | 100.00 |
|  |  | *29 | 1 | 1 | 100.00 |
|  |  | *01 | 2 | 2 | 100.00 |
|  |  | *30 | 4 | 4 | 100.00 |
|  |  | *03 | 1 | 1 | 100.00 |
|  |  | *32 | 1 | 1 | 100.00 |
|  | **Indian^#^** | *02 | 116 | 105 | 90.52 |
|  |  | *24 | 116 | 115 | 99.14 |
|  |  | *11 | 80 | 79 | 98.75 |
|  |  | *01 | 100 | 99 | 99.00 |
|  |  | *33 | 40 | 39 | 97.50 |
|  |  | *03 | 35 | 35 | 100.00 |
|  |  | *68 | 22 | 22 | 100.00 |
|  |  | *26 | 17 | 17 | 100.00 |
|  |  | *31 | 14 | 13 | 92.86 |
|  |  | *32 | 18 | 18 | 100.00 |
|  |  | *30 | 9 | 7 | 77.78 |
|  |  | *29 | 4 | 4 | 100.00 |
|  |  | *34 | 1 | 1 | 100.00 |
| ***HLA-B*** | **All^#^** | *15 | 671 | 625 | 93.14 |
|  |  | *40 | 319 | 303 | 94.98 |
|  |  | *35 | 264 | 250 | 94.70 |
|  |  | *51 | 223 | 210 | 94.17 |
|  |  | *18 | 224 | 218 | 97.32 |
|  |  | *58 | 187 | 179 | 95.72 |
|  |  | *13 | 144 | 136 | 94.44 |
|  |  | *07 | 114 | 107 | 93.86 |
|  |  | *52 | 134 | 118 | 88.06 |
|  |  | *44 | 160 | 145 | 90.63 |
|  |  | *46 | 107 | 100 | 93.46 |
|  |  | *38 | 112 | 88 | 78.57 |
|  |  | *27 | 80 | 72 | 90.00 |
|  |  | *57 | 76 | 75 | 98.68 |
|  |  | *37 | 47 | 41 | 87.23 |
|  |  | *55 | 64 | 57 | 89.06 |
|  |  | *08 | 27 | 24 | 88.89 |
|  |  | *39 | 32 | 9 | 28.13 |
|  |  | *48 | 20 | 19 | 95.00 |
|  |  | *54 | 13 | 11 | 84.62 |
|  | **Malay** | *15 | 516 | 493 | 95.54 |
|  |  | *18 | 211 | 205 | 97.16 |
|  |  | *35 | 169 | 161 | 95.27 |
|  |  | *40 | 126 | 118 | 93.65 |
|  |  | *51 | 118 | 111 | 94.07 |
|  |  | *44 | 121 | 115 | 95.04 |
|  |  | *58 | 113 | 107 | 94.69 |
|  |  | *13 | 81 | 79 | 97.53 |
|  |  | *52 | 79 | 70 | 88.61 |
|  |  | *07 | 70 | 67 | 95.71 |
|  |  | *38 | 77 | 56 | 72.73 |
|  |  | *27 | 59 | 57 | 96.61 |
|  |  | *46 | 50 | 45 | 90.00 |
|  |  | *37 | 15 | 13 | 86.67 |
|  |  | *55 | 24 | 21 | 87.50 |
|  |  | *57 | 20 | 20 | 100.00 |
|  |  | *39 | 16 | 5 | 31.25 |
|  |  | *48 | 13 | 12 | 92.31 |
|  |  | *08 | 9 | 8 | 88.89 |
|  |  | *54 | 4 | 2 | 50.00 |
|  | **Chinese** | *40 | 84 | 81 | 96.43 |
|  |  | *46 | 53 | 51 | 96.23 |
|  |  | *15 | 54 | 43 | 79.63 |
|  |  | *13 | 36 | 33 | 91.67 |
|  |  | *58 | 46 | 45 | 97.83 |
|  |  | *38 | 22 | 22 | 100.00 |
|  |  | *55 | 23 | 21 | 91.30 |
|  |  | *51 | 21 | 18 | 85.71 |
|  |  | *35 | 14 | 12 | 85.71 |
|  |  | *27 | 9 | 7 | 77.78 |
|  |  | *54 | 7 | 7 | 100.00 |
|  |  | *39 | 7 | 4 | 57.14 |
|  |  | *52 | 9 | 3 | 33.33 |
|  |  | *48 | 4 | 4 | 100.00 |
|  |  | *44 | 3 | 3 | 100.00 |
|  |  | *07 | 1 | 1 | 100.00 |
|  |  | *18 | 1 | 1 | 100.00 |
|  |  | *37 | 1 | 1 | 100.00 |
|  |  | *08 | 1 | 1 | 100.00 |
|  |  | *67 | 1 | 1 | 100.00 |
|  |  | *50 | 1 | 1 | 100.00 |
|  |  | *57 | 1 | 1 | 100.00 |
|  | **Indian** | *40 | 89 | 86 | 96.63 |
|  |  | *51 | 73 | 70 | 95.89 |
|  |  | *35 | 64 | 61 | 95.31 |
|  |  | *15 | 55 | 44 | 80.00 |
|  |  | *07 | 37 | 34 | 91.89 |
|  |  | *57 | 53 | 52 | 98.11 |
|  |  | *37 | 28 | 24 | 85.71 |
|  |  | *58 | 18 | 17 | 94.44 |
|  |  | *44 | 27 | 20 | 74.07 |
|  |  | *13 | 20 | 17 | 85.00 |
|  |  | *55 | 15 | 13 | 86.67 |
|  |  | *27 | 7 | 4 | 57.14 |
|  |  | *38 | 5 | 5 | 100.00 |
|  |  | *48 | 3 | 3 | 100.00 |
|  |  | *46 | 1 | 1 | 100.00 |
| ***HLA-C*** | **All^#^** | *07 | 812 | 787 | 96.92 |
|  |  | *04 | 508 | 488 | 96.06 |
|  |  | *03 | 430 | 415 | 96.51 |
|  |  | *08 | 427 | 398 | 93.21 |
|  |  | *12 | 215 | 208 | 96.74 |
|  |  | *01 | 204 | 193 | 94.61 |
|  |  | *06 | 171 | 156 | 91.23 |
|  |  | *15 | 154 | 125 | 81.17 |
|  |  | *14 | 149 | 142 | 95.30 |
|  |  | *16 | 35 | 32 | 91.43 |
|  | **Malay** | *07 | 573 | 556 | 97.03 |
|  |  | *04 | 370 | 357 | 96.49 |
|  |  | *08 | 342 | 320 | 93.57 |
|  |  | *03 | 234 | 228 | 97.44 |
|  |  | *12 | 100 | 94 | 94.00 |
|  |  | *01 | 91 | 84 | 92.31 |
|  |  | *14 | 90 | 85 | 94.44 |
|  |  | *15 | 66 | 49 | 74.24 |
|  |  | *06 | 57 | 55 | 96.49 |
|  |  | *16 | 12 | 12 | 100.00 |
|  | **Chinese** | *03 | 136 | 132 | 97.06 |
|  |  | *07 | 81 | 79 | 97.53 |
|  |  | *01 | 79 | 78 | 98.73 |
|  |  | *08 | 39 | 38 | 97.44 |
|  |  | *04 | 22 | 20 | 90.91 |
|  |  | *12 | 20 | 20 | 100.00 |
|  |  | *15 | 12 | 11 | 91.67 |
|  |  | *14 | 11 | 10 | 90.91 |
|  |  | *06 | 9 | 7 | 77.78 |
|  |  | *16 | 3 | 2 | 66.67 |
|  | **Indian** | *07 | 107 | 103 | 96.26 |
|  |  | *06 | 97 | 86 | 88.66 |
|  |  | *12 | 83 | 82 | 98.80 |
|  |  | *04 | 80 | 76 | 95.00 |
|  |  | *15 | 62 | 54 | 87.10 |
|  |  | *03 | 37 | 35 | 94.59 |
|  |  | *14 | 41 | 41 | 100.00 |
|  |  | *01 | 29 | 26 | 89.66 |
|  |  | *08 | 17 | 13 | 76.47 |
|  |  | *16 | 20 | 18 | 90.00 |
| ***HLA-DRB1*** | **All^#^** | *15 | 750 | 658 | 87.73 |
|  |  | *12 | 673 | 639 | 94.95 |
|  |  | *04 | 265 | 252 | 95.09 |
|  |  | *07 | 310 | 290 | 93.55 |
|  |  | *14 | 204 | 192 | 94.12 |
|  |  | *03 | 196 | 182 | 92.86 |
|  |  | *10 | 95 | 86 | 90.53 |
|  |  | *09 | 133 | 122 | 91.73 |
|  |  | *16 | 124 | 113 | 91.13 |
|  |  | *13 | 143 | 123 | 86.01 |
|  |  | *11 | 112 | 93 | 83.04 |
|  |  | *08 | 102 | 92 | 90.20 |
|  |  | *01 | 33 | 28 | 84.85 |
|  | **Malay** | *12 | 562 | 543 | 96.62 |
|  |  | *15 | 516 | 440 | 85.27 |
|  |  | *04 | 128 | 122 | 95.31 |
|  |  | *07 | 196 | 181 | 92.35 |
|  |  | *03 | 103 | 95 | 92.23 |
|  |  | *14 | 91 | 83 | 91.21 |
|  |  | *16 | 78 | 72 | 92.31 |
|  |  | *09 | 63 | 57 | 90.48 |
|  |  | *10 | 37 | 33 | 89.19 |
|  |  | *13 | 65 | 55 | 84.62 |
|  |  | *11 | 63 | 51 | 80.95 |
|  |  | *08 | 47 | 42 | 89.36 |
|  |  | *01 | 13 | 10 | 76.92 |
|  | **Chinese** | *04 | 45 | 45 | 100.00 |
|  |  | *15 | 56 | 50 | 89.29 |
|  |  | *09 | 58 | 56 | 96.55 |
|  |  | *12 | 60 | 51 | 85.00 |
|  |  | *16 | 34 | 32 | 94.12 |
|  |  | *14 | 43 | 41 | 95.35 |
|  |  | *08 | 31 | 30 | 96.77 |
|  |  | *03 | 41 | 39 | 95.12 |
|  |  | *11 | 18 | 16 | 88.89 |
|  |  | *13 | 11 | 6 | 54.55 |
|  |  | *10 | 2 | 2 | 100.00 |
|  |  | *07 | 11 | 10 | 90.91 |
|  |  | *01 | 2 | 1 | 50.00 |
|  | **Indian** | *15 | 129 | 123 | 95.35 |
|  |  | *04 | 77 | 70 | 90.91 |
|  |  | *07 | 89 | 86 | 96.63 |
|  |  | *10 | 52 | 47 | 90.38 |
|  |  | *14 | 58 | 58 | 100.00 |
|  |  | *13 | 62 | 58 | 93.55 |
|  |  | *03 | 31 | 30 | 96.77 |
|  |  | *12 | 20 | 15 | 75.00 |
|  |  | *11 | 26 | 22 | 84.62 |
|  |  | *08 | 17 | 13 | 76.47 |
|  |  | *01 | 13 | 12 | 92.31 |
|  |  | *09 | 3 | 2 | 66.67 |
|  |  | *16 | 3 | 2 | 66.67 |
| ***HLA-DQB1*** | **All^#^** | *03 | 1132 | 1108 | 95.5 |
|  |  | *05 | 915 | 878 | 96.0 |
|  |  | *06 | 553 | 393 | 71.1 |
|  |  | *02 | 423 | 404 | 95.5 |
|  |  | *04 | 91 | 88 | 96.7 |
|  | **Malay** | *03 | 732 | 717 | 98.4 |
|  |  | *05 | 606 | 581 | 96.8 |
|  |  | *06 | 287 | 157 | 58.1 |
|  |  | *02 | 269 | 257 | 96.6 |
|  |  | *04 | 54 | 51 | 96.2 |
|  | **Chinese** | *03 | 164 | 162 | 98.8 |
|  |  | *05 | 102 | 100 | 98.0 |
|  |  | *06 | 68 | 66 | 97.1 |
|  |  | *04 | 20 | 20 | 100.0 |
|  |  | *02 | 52 | 50 | 96.15 |
|  | **Indian** | *03 | 174 | 171 | 98.3 |
|  |  | *05 | 148 | 143 | 96.6 |
|  |  | *06 | 171 | 147 | 86.0 |
|  |  | *02 | 70 | 68 | 97.1 |
|  |  | *04 | 10 | 10 | 100.0 |

Footnote: Imputation accuracy of each imputed classical 2-digit HLA alleles for *HLA-A, HLA-B, HLA-C, HLA-DRB1* and *HLA-DQB1* genes in the Malays, Chinese and Indians. All: combined group of individuals with Malay, Chinese, Indian and other/mixed ethnicities. ^a^HLA alleles with available data for both lab-based and computational-imputed HLA allele frequency. Alleles arranged in descending order of allelic frequency of experimental classical HLA allele frequency. Concordance rate is the total number of imputed classical HLA alleles concordant with experimental classical HLA alleles divided by the total number of detected experimental classical HLA alleles within the population. ^#^Only individuals with available data were included in this analysis.

**Supplementary table 3** Classical 4-digit HLA alleles imputation accuracy in the control group, stratified by ethnicity

| **HLA genes** | **Ethnicity** | **HLA alleles**^a^ | **gHLA allele count** | **Matched iHLA allele count** | **Concordance rate (%)** |
| --- | --- | --- | --- | --- | --- |
| ***HLA-A*** | **All** | *24:02 | 578 | 456 | 78.89 |
|  |  | *11:01 | 572 | 531 | 92.83 |
|  |  | *24:07 | 297 | 252 | 84.85 |
|  |  | *02:01 | 236 | 135 | 57.20 |
|  |  | *01:01 | 148 | 145 | 97.97 |
|  |  | *34:01 | 127 | 121 | 95.28 |
|  |  | *33:03 | 100 | 99 | 99.00 |
|  |  | *02:03 | 74 | 56 | 75.68 |
|  |  | *02:06 | 68 | 66 | 97.06 |
|  |  | *02:11 | 67 | 61 | 91.04 |
|  |  | *26:01 | 63 | 60 | 95.24 |
|  |  | *24:10 | 55 | 49 | 89.09 |
|  |  | *03:01 | 51 | 49 | 96.08 |
|  |  | *68:01 | 47 | 45 | 95.74 |
|  |  | *31:01 | 28 | 28 | 100.00 |
|  |  | *32:01 | 28 | 28 | 100.00 |
|  |  | *30:01 | 25 | 22 | 88.00 |
|  |  | *02:07 | 20 | 20 | 100.00 |
|  |  | *29:01 | 15 | 15 | 100.00 |
|  |  | *11:02 | 5 | 5 | 100.00 |
|  | **Malay** | *24:02 | 390 | 285 | 73.08 |
|  |  | *11:01 | 334 | 310 | 92.81 |
|  |  | *24:07 | 265 | 233 | 87.92 |
|  |  | *02:01 | 122 | 94 | 77.05 |
|  |  | *34:01 | 117 | 111 | 94.87 |
|  |  | *33:03 | 76 | 76 | 100.00 |
|  |  | *24:10 | 51 | 46 | 90.20 |
|  |  | *01:01 | 47 | 45 | 95.74 |
|  |  | *02:03 | 38 | 26 | 68.42 |
|  |  | *02:06 | 35 | 35 | 100.00 |
|  |  | *26:01 | 31 | 29 | 93.55 |
|  |  | *68:01 | 23 | 23 | 100.00 |
|  |  | *03:01 | 20 | 18 | 90.00 |
|  |  | *02:11 | 15 | 13 | 86.67 |
|  |  | *30:01 | 13 | 12 | 92.31 |
|  |  | *32:01 | 8 | 8 | 100.00 |
|  |  | *02:07 | 8 | 8 | 100.00 |
|  |  | *29:01 | 8 | 8 | 100.00 |
|  |  | *31:01 | 7 | 7 | 100.00 |
|  |  | *11:02 | 2 | 2 | 100.00 |
|  | **Chinese** | *11:01 | 127 | 112 | 88.19 |
|  |  | *24:02 | 62 | 58 | 93.55 |
|  |  | *02:01 | 56 | 14 | 25.00 |
|  |  | *02:03 | 25 | 22 | 88.00 |
|  |  | *02:06 | 18 | 17 | 94.44 |
|  |  | *26:01 | 13 | 13 | 100.00 |
|  |  | *02:07 | 11 | 11 | 100.00 |
|  |  | *33:03 | 10 | 10 | 100.00 |
|  |  | *31:01 | 6 | 6 | 100.00 |
|  |  | *24:07 | 4 | 3 | 75.00 |
|  |  | *30:01 | 4 | 4 | 100.00 |
|  |  | *11:02 | 3 | 3 | 100.00 |
|  |  | *01:01 | 2 | 2 | 100.00 |
|  |  | *24:10 | 1 | 1 | 100.00 |
|  |  | *03:01 | 1 | 1 | 100.00 |
|  |  | *29:01 | 1 | 1 | 100.00 |
|  | **Indian** | *24:02 | 96 | 87 | 90.63 |
|  |  | *01:01 | 95 | 94 | 98.95 |
|  |  | *11:01 | 73 | 73 | 100.00 |
|  |  | *02:11 | 48 | 44 | 91.67 |
|  |  | *02:01 | 38 | 16 | 42.11 |
|  |  | *03:01 | 28 | 28 | 100.00 |
|  |  | *68:01 | 21 | 20 | 95.24 |
|  |  | *32:01 | 18 | 18 | 100.00 |
|  |  | *26:01 | 16 | 16 | 100.00 |
|  |  | *02:06 | 12 | 11 | 91.67 |
|  |  | *31:01 | 12 | 12 | 100.00 |
|  |  | *24:07 | 10 | 1 | 10.00 |
|  |  | *33:03 | 10 | 9 | 90.00 |
|  |  | *30:01 | 7 | 5 | 71.43 |
|  |  | *29:01 | 4 | 4 | 100.00 |
|  |  | *02:03 | 3 | 2 | 66.67 |
|  |  | *34:01 | 1 | 1 | 100.00 |
| ***HLA-B*** | **All** | *15:02 | 282 | 241 | 85.46 |
|  |  | *58:01 | 184 | 179 | 97.28 |
|  |  | *44:03 | 155 | 141 | 90.97 |
|  |  | *40:01 | 148 | 129 | 87.16 |
|  |  | *51:01 | 145 | 140 | 96.55 |
|  |  | *18:01 | 140 | 137 | 97.86 |
|  |  | *15:13 | 138 | 122 | 88.41 |
|  |  | *52:01 | 130 | 119 | 91.54 |
|  |  | *13:01 | 124 | 107 | 86.29 |
|  |  | *35:05 | 111 | 97 | 87.39 |
|  |  | *38:02 | 107 | 85 | 79.44 |
|  |  | *46:01 | 106 | 98 | 92.45 |
|  |  | *15:21 | 88 | 75 | 85.23 |
|  |  | *35:01 | 86 | 31 | 36.05 |
|  |  | *40:06 | 81 | 68 | 83.95 |
|  |  | *57:01 | 76 | 74 | 97.37 |
|  |  | *07:05 | 75 | 64 | 85.33 |
|  |  | *27:06 | 55 | 49 | 89.09 |
|  |  | *35:03 | 51 | 40 | 78.43 |
|  |  | *15:25 | 49 | 32 | 65.31 |
|  |  | *37:01 | 46 | 43 | 93.48 |
|  |  | *07:02 | 35 | 31 | 88.57 |
|  |  | *55:02 | 30 | 26 | 86.67 |
|  |  | *15:01 | 28 | 22 | 78.57 |
|  |  | *40:02 | 27 | 9 | 33.33 |
|  |  | *08:01 | 26 | 21 | 80.77 |
|  |  | *39:01 | 26 | 8 | 30.77 |
|  |  | *15:18 | 19 | 17 | 89.47 |
|  |  | *27:04 | 19 | 13 | 68.42 |
|  |  | *50:01 | 18 | 15 | 83.33 |
|  |  | *13:02 | 17 | 15 | 88.24 |
|  |  | *48:01 | 16 | 16 | 100.00 |
|  |  | *54:01 | 13 | 11 | 84.62 |
|  |  | *67:01 | 1 | 1 | 100.00 |
|  | **Malay** | *15:02 | 230 | 199 | 86.52 |
|  |  | *18:01 | 130 | 127 | 97.69 |
|  |  | *15:13 | 119 | 108 | 90.76 |
|  |  | *44:03 | 118 | 111 | 94.07 |
|  |  | *58:01 | 112 | 108 | 96.43 |
|  |  | *35:05 | 100 | 90 | 90.00 |
|  |  | *15:21 | 80 | 69 | 86.25 |
|  |  | *52:01 | 78 | 72 | 92.31 |
|  |  | *51:01 | 74 | 72 | 97.30 |
|  |  | *38:02 | 74 | 55 | 74.32 |
|  |  | *40:01 | 70 | 59 | 84.29 |
|  |  | *13:01 | 69 | 61 | 88.41 |
|  |  | *46:01 | 50 | 44 | 88.00 |
|  |  | *07:05 | 48 | 41 | 85.42 |
|  |  | *27:06 | 48 | 45 | 93.75 |
|  |  | *35:01 | 47 | 7 | 14.89 |
|  |  | *15:25 | 38 | 24 | 63.16 |
|  |  | *40:06 | 26 | 20 | 76.92 |
|  |  | *57:01 | 20 | 20 | 100.00 |
|  |  | *07:02 | 19 | 16 | 84.21 |
|  |  | *37:01 | 15 | 13 | 86.67 |
|  |  | *35:03 | 13 | 11 | 84.62 |
|  |  | *40:02 | 13 | 5 | 38.46 |
|  |  | *39:01 | 12 | 5 | 41.67 |
|  |  | *48:01 | 11 | 11 | 100.00 |
|  |  | *55:02 | 10 | 9 | 90.00 |
|  |  | *13:02 | 10 | 9 | 90.00 |
|  |  | *08:01 | 9 | 8 | 88.89 |
|  |  | *27:04 | 8 | 4 | 50.00 |
|  |  | *15:01 | 7 | 5 | 71.43 |
|  |  | *15:18 | 5 | 4 | 80.00 |
|  |  | *50:01 | 5 | 2 | 40.00 |
|  |  | *54:01 | 4 | 3 | 75.00 |
|  | **Chinese** | *46:01 | 52 | 50 | 96.15 |
|  |  | *40:01 | 51 | 48 | 94.12 |
|  |  | *58:01 | 46 | 46 | 100.00 |
|  |  | *13:01 | 32 | 29 | 90.63 |
|  |  | *15:02 | 24 | 20 | 83.33 |
|  |  | *38:02 | 20 | 20 | 100.00 |
|  |  | *55:02 | 19 | 16 | 84.21 |
|  |  | *51:01 | 10 | 8 | 80.00 |
|  |  | *52:01 | 9 | 4 | 44.44 |
|  |  | *40:06 | 9 | 7 | 77.78 |
|  |  | *15:01 | 9 | 5 | 55.56 |
|  |  | *35:01 | 7 | 6 | 85.71 |
|  |  | *40:02 | 7 | 4 | 57.14 |
|  |  | *27:04 | 7 | 5 | 71.43 |
|  |  | *54:01 | 7 | 6 | 85.71 |
|  |  | *15:25 | 6 | 4 | 66.67 |
|  |  | *39:01 | 6 | 3 | 50.00 |
|  |  | *35:05 | 4 | 1 | 25.00 |
|  |  | *48:01 | 4 | 4 | 100.00 |
|  |  | *44:03 | 3 | 3 | 100.00 |
|  |  | *13:02 | 3 | 3 | 100.00 |
|  |  | *15:13 | 2 | 1 | 50.00 |
|  |  | *27:06 | 2 | 2 | 100.00 |
|  |  | *15:18 | 2 | 1 | 50.00 |
|  |  | *57:01 | 1 | 1 | 100.00 |
|  |  | *07:05 | 1 | 1 | 100.00 |
|  |  | *35:03 | 1 | 1 | 100.00 |
|  |  | *37:01 | 1 | 1 | 100.00 |
|  |  | *08:01 | 1 | 1 | 100.00 |
|  |  | *50:01 | 1 | 1 | 100.00 |
|  |  | *67:01 | 1 | 1 | 100.00 |
|  | **Indian** | *51:01 | 56 | 55 | 98.21 |
|  |  | *57:01 | 53 | 51 | 96.23 |
|  |  | *40:06 | 41 | 38 | 92.68 |
|  |  | *52:01 | 35 | 35 | 100.00 |
|  |  | *35:03 | 33 | 26 | 78.79 |
|  |  | *37:01 | 27 | 26 | 96.30 |
|  |  | *35:01 | 26 | 14 | 53.85 |
|  |  | *44:03 | 25 | 20 | 80.00 |
|  |  | *07:05 | 21 | 19 | 90.48 |
|  |  | *15:02 | 18 | 13 | 72.22 |
|  |  | *58:01 | 17 | 16 | 94.12 |
|  |  | *40:01 | 17 | 13 | 76.47 |
|  |  | *13:01 | 16 | 11 | 68.75 |
|  |  | *07:02 | 16 | 15 | 93.75 |
|  |  | *15:18 | 11 | 11 | 100.00 |
|  |  | *08:01 | 10 | 8 | 80.00 |
|  |  | *50:01 | 10 | 10 | 100.00 |
|  |  | *18:01 | 7 | 7 | 100.00 |
|  |  | *15:01 | 7 | 7 | 100.00 |
|  |  | *38:02 | 5 | 5 | 100.00 |
|  |  | *13:02 | 4 | 3 | 75.00 |
|  |  | *27:04 | 3 | 3 | 100.00 |
|  |  | *27:06 | 2 | 1 | 50.00 |
|  |  | *15:25 | 2 | 2 | 100.00 |
|  |  | *46:01 | 1 | 1 | 100.00 |
|  |  | *48:01 | 1 | 1 | 100.00 |
| ***HLA-C*** | **All** | *08:01 | 402 | 388 | 96.52 |
|  |  | *07:02 | 349 | 332 | 95.13 |
|  |  | *04:01 | 287 | 273 | 95.12 |
|  |  | *07:01 | 212 | 183 | 86.32 |
|  |  | *07:04 | 206 | 192 | 93.20 |
|  |  | *03:02 | 198 | 170 | 85.86 |
|  |  | *01:02 | 182 | 175 | 96.15 |
|  |  | *04:03 | 176 | 167 | 94.89 |
|  |  | *06:02 | 169 | 160 | 94.67 |
|  |  | *12:02 | 152 | 139 | 91.45 |
|  |  | *14:02 | 146 | 139 | 95.21 |
|  |  | *03:04 | 138 | 131 | 94.93 |
|  |  | *15:02 | 130 | 121 | 93.08 |
|  |  | *03:03 | 74 | 59 | 79.73 |
|  |  | *12:03 | 58 | 57 | 98.28 |
|  |  | *16:02 | 32 | 29 | 90.63 |
|  |  | *15:05 | 7 | 6 | 85.71 |
|  | **Malay** | *08:01 | 321 | 314 | 97.82 |
|  |  | *07:02 | 215 | 205 | 95.35 |
|  |  | *04:01 | 193 | 182 | 94.30 |
|  |  | *07:04 | 172 | 163 | 94.77 |
|  |  | *07:01 | 158 | 144 | 91.14 |
|  |  | *04:03 | 139 | 133 | 95.68 |
|  |  | *03:02 | 119 | 105 | 88.24 |
|  |  | *14:02 | 87 | 82 | 94.25 |
|  |  | *03:04 | 80 | 77 | 96.25 |
|  |  | *01:02 | 79 | 75 | 94.94 |
|  |  | *12:02 | 74 | 70 | 94.59 |
|  |  | *06:02 | 56 | 55 | 98.21 |
|  |  | *15:02 | 53 | 47 | 88.68 |
|  |  | *03:03 | 26 | 22 | 84.62 |
|  |  | *12:03 | 24 | 23 | 95.83 |
|  |  | *16:02 | 11 | 11 | 100.00 |
|  |  | *15:05 | 5 | 4 | 80.00 |
|  | **Chinese** | *01:02 | 72 | 71 | 98.61 |
|  |  | *07:02 | 62 | 60 | 96.77 |
|  |  | *03:02 | 49 | 40 | 81.63 |
|  |  | *03:04 | 46 | 42 | 91.30 |
|  |  | *08:01 | 37 | 36 | 97.30 |
|  |  | *03:03 | 31 | 23 | 74.19 |
|  |  | *04:01 | 11 | 10 | 90.91 |
|  |  | *12:02 | 11 | 11 | 100.00 |
|  |  | *14:02 | 11 | 10 | 90.91 |
|  |  | *15:02 | 11 | 11 | 100.00 |
|  |  | *04:03 | 10 | 9 | 90.00 |
|  |  | *07:01 | 9 | 3 | 33.33 |
|  |  | *06:02 | 9 | 8 | 88.89 |
|  |  | *12:03 | 9 | 9 | 100.00 |
|  |  | *07:04 | 3 | 2 | 66.67 |
|  |  | *16:02 | 2 | 1 | 50.00 |
|  | **Indian** | *06:02 | 96 | 89 | 92.71 |
|  |  | *04:01 | 62 | 60 | 96.77 |
|  |  | *12:02 | 59 | 50 | 84.75 |
|  |  | *15:02 | 55 | 52 | 94.55 |
|  |  | *07:02 | 48 | 44 | 91.67 |
|  |  | *14:02 | 41 | 41 | 100.00 |
|  |  | *07:01 | 34 | 27 | 79.41 |
|  |  | *01:02 | 26 | 24 | 92.31 |
|  |  | *12:03 | 21 | 21 | 100.00 |
|  |  | *03:02 | 20 | 16 | 80.00 |
|  |  | *16:02 | 19 | 17 | 89.47 |
|  |  | *07:04 | 18 | 16 | 88.89 |
|  |  | *08:01 | 16 | 12 | 75.00 |
|  |  | *04:03 | 15 | 13 | 86.67 |
|  |  | *03:03 | 9 | 9 | 100.00 |
|  |  | *03:04 | 8 | 8 | 100.00 |
|  |  | *15:05 | 1 | 1 | 100.00 |
| ***HLA-DRB1*** | **All** | *15:02 | 455 | 398 | 87.47 |
|  |  | *12:02 | 369 | 351 | 95.12 |
|  |  | *12:01 | 302 | 11 | 3.64 |
|  |  | *07:01 | 280 | 262 | 93.57 |
|  |  | *15:01 | 246 | 172 | 69.92 |
|  |  | *03:01 | 171 | 164 | 95.91 |
|  |  | *09:01 | 129 | 120 | 93.02 |
|  |  | *16:02 | 119 | 106 | 89.08 |
|  |  | *14:04 | 115 | 94 | 81.74 |
|  |  | *04:03 | 97 | 83 | 85.57 |
|  |  | *04:05 | 96 | 80 | 83.33 |
|  |  | *10:01 | 91 | 82 | 90.11 |
|  |  | *11:01 | 84 | 78 | 92.86 |
|  |  | *08:03 | 78 | 72 | 92.31 |
|  |  | *13:01 | 67 | 53 | 79.10 |
|  |  | *14:01 | 61 | 33 | 54.10 |
|  |  | *13:02 | 60 | 51 | 85.00 |
|  |  | *01:01 | 27 | 22 | 81.48 |
|  |  | *04:06 | 24 | 19 | 79.17 |
|  |  | *04:01 | 21 | 14 | 66.67 |
|  |  | *14:05 | 13 | 3 | 23.08 |
|  |  | *08:02 | 7 | 4 | 57.14 |
|  |  | *13:12 | 4 | 4 | 100.00 |
|  | **Malay** | *15:02 | 357 | 304 | 85.15 |
|  |  | *12:02 | 312 | 299 | 95.83 |
|  |  | *12:01 | 248 | 1 | 0.40 |
|  |  | *07:01 | 179 | 165 | 92.18 |
|  |  | *15:01 | 125 | 62 | 49.60 |
|  |  | *03:01 | 94 | 91 | 96.81 |
|  |  | *16:02 | 76 | 68 | 89.47 |
|  |  | *04:05 | 68 | 56 | 82.35 |
|  |  | *09:01 | 61 | 56 | 91.80 |
|  |  | *14:04 | 61 | 46 | 75.41 |
|  |  | *11:01 | 46 | 42 | 91.30 |
|  |  | *10:01 | 37 | 33 | 89.19 |
|  |  | *08:03 | 35 | 30 | 85.71 |
|  |  | *04:03 | 32 | 29 | 90.63 |
|  |  | *13:01 | 32 | 20 | 62.50 |
|  |  | *13:02 | 30 | 24 | 80.00 |
|  |  | *14:01 | 23 | 14 | 60.87 |
|  |  | *04:06 | 11 | 7 | 63.64 |
|  |  | *01:01 | 10 | 7 | 70.00 |
|  |  | *04:01 | 4 | 4 | 100.00 |
|  |  | *08:02 | 3 | 2 | 66.67 |
|  | **Chinese** | *09:01 | 58 | 56 | 96.55 |
|  |  | *12:01 | 38 | 8 | 21.05 |
|  |  | *03:01 | 35 | 33 | 94.29 |
|  |  | *15:01 | 33 | 31 | 93.94 |
|  |  | *16:02 | 33 | 31 | 93.94 |
|  |  | *08:03 | 27 | 27 | 100.00 |
|  |  | *14:01 | 25 | 17 | 68.00 |
|  |  | *12:02 | 22 | 19 | 86.36 |
|  |  | *15:02 | 17 | 16 | 94.12 |
|  |  | *04:05 | 17 | 17 | 100.00 |
|  |  | *11:01 | 14 | 14 | 100.00 |
|  |  | *04:03 | 12 | 5 | 41.67 |
|  |  | *07:01 | 10 | 9 | 90.00 |
|  |  | *04:06 | 10 | 9 | 90.00 |
|  |  | *14:05 | 10 | 3 | 30.00 |
|  |  | *13:02 | 6 | 6 | 100.00 |
|  |  | *14:04 | 5 | 3 | 60.00 |
|  |  | *13:12 | 3 | 3 | 100.00 |
|  |  | *10:01 | 2 | 2 | 100.00 |
|  |  | *01:01 | 2 | 1 | 50.00 |
|  |  | *04:01 | 2 | 2 | 100.00 |
|  | **Indian** | *07:01 | 77 | 75 | 97.40 |
|  |  | *15:01 | 71 | 66 | 92.96 |
|  |  | *15:02 | 53 | 51 | 96.23 |
|  |  | *10:01 | 49 | 44 | 89.80 |
|  |  | *04:03 | 46 | 42 | 91.30 |
|  |  | *14:04 | 43 | 40 | 93.02 |
|  |  | *13:01 | 34 | 33 | 97.06 |
|  |  | *03:01 | 25 | 24 | 96.00 |
|  |  | *13:02 | 20 | 17 | 85.00 |
|  |  | *11:01 | 19 | 17 | 89.47 |
|  |  | *04:01 | 15 | 8 | 53.33 |
|  |  | *12:02 | 13 | 11 | 84.62 |
|  |  | *08:03 | 11 | 10 | 90.91 |
|  |  | *01:01 | 11 | 10 | 90.91 |
|  |  | *12:01 | 7 | 1 | 14.29 |
|  |  | *04:05 | 5 | 1 | 20.00 |
|  |  | *09:01 | 3 | 2 | 66.67 |
|  |  | *08:02 | 3 | 2 | 66.67 |
|  |  | *04:06 | 2 | 2 | 100.00 |
| ***HLA-DQB1*** | **All** | *03:01 | 758 | 734 | 96.83 |
|  |  | *06:01 | 376 | 214 | 56.91 |
|  |  | *05:02 | 372 | 341 | 91.67 |
|  |  | *05:01 | 360 | 335 | 93.06 |
|  |  | *02:01 | 221 | 214 | 96.83 |
|  |  | *03:03 | 195 | 187 | 95.90 |
|  |  | *05:03 | 186 | 181 | 97.31 |
|  |  | *03:02 | 177 | 163 | 92.09 |
|  |  | *06:03 | 62 | 61 | 98.39 |
|  |  | *04:02 | 47 | 45 | 95.74 |
|  |  | *04:01 | 44 | 39 | 88.64 |
|  |  | *06:02 | 41 | 40 | 97.56 |
|  |  | *06:09 | 38 | 36 | 94.74 |
|  |  | *06:04 | 13 | 12 | 92.31 |
|  | **Malay** | *03:01 | 581 | 567 | 97.59 |
|  |  | *05:02 | 267 | 248 | 92.88 |
|  |  | *05:01 | 251 | 234 | 93.23 |
|  |  | *06:01 | 202 | 73 | 36.14 |
|  |  | *02:01 | 128 | 124 | 96.88 |
|  |  | *05:03 | 88 | 83 | 94.32 |
|  |  | *03:03 | 74 | 70 | 94.59 |
|  |  | *03:02 | 74 | 68 | 91.89 |
|  |  | *04:02 | 28 | 27 | 96.43 |
|  |  | *04:01 | 26 | 22 | 84.62 |
|  |  | *06:03 | 25 | 24 | 96.00 |
|  |  | *06:09 | 22 | 21 | 95.45 |
|  |  | *06:02 | 21 | 20 | 95.24 |
|  |  | *06:04 | 5 | 4 | 80.00 |
|  | **Chinese** | *03:01 | 77 | 72 | 93.51 |
|  |  | *05:02 | 66 | 63 | 95.45 |
|  |  | *03:03 | 61 | 61 | 100.00 |
|  |  | *06:01 | 53 | 49 | 92.45 |
|  |  | *02:01 | 42 | 40 | 95.24 |
|  |  | *03:02 | 29 | 27 | 93.10 |
|  |  | *05:03 | 21 | 21 | 100.00 |
|  |  | *05:01 | 16 | 15 | 93.75 |
|  |  | *04:01 | 14 | 14 | 100.00 |
|  |  | *04:02 | 6 | 5 | 83.33 |
|  |  | *06:02 | 5 | 5 | 100.00 |
|  |  | *06:09 | 4 | 4 | 100.00 |
|  | **Indian** | *06:01 | 101 | 79 | 78.22 |
|  |  | *05:01 | 68 | 63 | 92.65 |
|  |  | *05:03 | 67 | 67 | 100.00 |
|  |  | *03:03 | 63 | 59 | 93.65 |
|  |  | *03:02 | 62 | 59 | 95.16 |
|  |  | *04:01 | 50 | 48 | 96.00 |
|  |  | *06:03 | 37 | 37 | 100.00 |
|  |  | *02:01 | 31 | 31 | 100.00 |
|  |  | *05:02 | 16 | 11 | 68.75 |
|  |  | *04:02 | 10 | 10 | 100.00 |
|  |  | *06:02 | 10 | 10 | 100.00 |
|  |  | *06:09 | 9 | 8 | 88.89 |
|  |  | *06:04 | 7 | 7 | 100.00 |

Footnote: Imputation accuracy of each imputed classical 4-digit HLA alleles for *HLA-A, HLA-B, HLA-C, HLA-DRB1* and *HLA-DQB1* genes in the Malays, Chinese and Indians. All: combined group of individuals with Malay, Chinese, Indian and other/mixed ethnicities. ^a^HLA alleles with available data for both lab-based and computational-imputed HLA allele frequency. Alleles arranged in descending order of allelic frequency of experimental classical HLA allele frequency. Concordance rate is the total number of imputed classical HLA alleles concordant with experimental classical HLA alleles divided by the total number of detected experimental classical HLA alleles within the population. ^#^Only individuals with available data were included in this analysis.

**Supplementary table 4. Number of imputed classical HLA alleles and amino acid polymorphisms achieved published genome-wide threshold of p<5x10^-08^**

| **HLA genes** | **Classical HLA alleles**  **(n=15)** | **Polymorphic amino acid residues (n=74)** |
| --- | --- | --- |
| ***HLA-A*** | *na* | 1 (1.4%) |
| ***HLA-B*** | *na* | 2 (2.7%) |
| ***HLA-C*** | *na* | *na* |
| ***HLA-DRB1*** | 6 (0.40%) | 35 (47.3%) |
| ***HLA-DQA1*** | 4 (26.7%) | 15 (20.3%) |
| ***HLA-DQB1*** | 5 (33.3%) | 21 (24.4%) |
| ***HLA-DPA1*** | *na* | *na* |
| ***HLA-DPB1*** | *na* | *na* |

Footnote: The data for classical HLA alleles included both classical HLA alleles at 2-digits and 4-digits resolution. na: no HLA variants achieved GWAS threshold

**Supplementary table 5.** Logistic regression results of the association between imputed HLA amino acids and alleles, and risk of developing ACPA-positive rheumatoid arthritis in the Malay, Chinese and Indian ethnic groups.

| **HLA variants** | **BP** | **Allele Frequency** | | **OR** | **95% CI** | **P** | **P-adjusted** |
| --- | --- | --- | --- | --- | --- | --- | --- |
|  |  | **RA cases** | **Controls** |  |  |  |  |
| **Malay** | | | | | | | |
| AA_DRB1_11_32660115_V | 32660115 | 0.28 | 0.08 | 4.26 | 3.30 - 5.49 | 7.22E-29 | 3.542E-25 |
| AA_DRB1_120_32657518_N | 32657518 | 0.28 | 0.09 | 4.23 | 3.28 - 5.45 | 8.59E-29 | 3.542E-25 |
| HLA-DRB1*04:05 | 32660042 | 0.15 | 0.03 | 6.09 | 4.28 - 8.67 | 1.26E-23 | 2.59E-20 |
| AA_DQB1_71_32740624_D | 32740624 | 0.15 | 0.03 | 6.27 | 4.36 - 9.01 | 3.11E-23 | 2.848E-20 |
| AA_DQB1_70_32740627_E | 32740627 | 0.15 | 0.03 | 6.27 | 4.36 - 9.01 | 3.11E-23 | 2.848E-20 |
| AA_DQB1_56_32740669 | 32740669 | 0.15 | 0.03 | 6.27 | 4.36 - 9.01 | 3.11E-23 | 2.848E-20 |
| AA_DRB1_37_32660037_Y | 32660037 | 0.30 | 0.13 | 2.78 | 2.22 - 3.48 | 6.16E-19 | 4.614E-16 |
| AA_DRB1_33_32660049 | 32660049 | 0.19 | 0.07 | 3.48 | 2.62 - 4.61 | 4.39E-18 | 2.26E-15 |
| AA_DRB1_13_32660109_H | 32660109 | 0.19 | 0.07 | 3.48 | 2.62 - 4.61 | 4.39E-18 | 2.26E-15 |
| AA_DRB1_-24_32665481_F | 32665481 | 0.19 | 0.07 | 3.48 | 2.62 - 4.61 | 4.39E-18 | 2.26E-15 |
| AA_DQB1_9_32740810_F | 32740810 | 0.15 | 0.04 | 4.17 | 3.02 - 5.76 | 5.53E-18 | 2.452E-15 |
| AA_DRB1_180_32657338_L | 32657338 | 0.19 | 0.07 | 3.44 | 2.60 - 4.55 | 5.65E-18 | 2.452E-15 |
| AA_DRB1_96_32657590_Y | 32657590 | 0.19 | 0.07 | 3.44 | 2.60 - 4.55 | 5.65E-18 | 2.452E-15 |
| AA_DRB1_57_32659977_S | 32659977 | 0.16 | 0.05 | 3.71 | 2.73 - 5.05 | 6.02E-17 | 2.362E-14 |
| HLA-DQA1*03:01 | 32716284 | 0.22 | 0.10 | 2.75 | 2.14 - 3.54 | 2.81E-15 | 7.241E-13 |
| AA_DQA1_26_32717128 | 32717128 | 0.22 | 0.10 | 2.75 | 2.14 - 3.54 | 2.81E-15 | 7.241E-13 |
| AA_DQA1_47_32717191_Q | 32717191 | 0.22 | 0.10 | 2.75 | 2.14 - 3.54 | 2.81E-15 | 7.241E-13 |
| AA_DQA1_56_32717218_R | 32717218 | 0.22 | 0.10 | 2.75 | 2.14 - 3.54 | 2.81E-15 | 7.241E-13 |
| AA_DQA1_76_32717278_V | 32717278 | 0.22 | 0.10 | 2.75 | 2.14 - 3.54 | 2.81E-15 | 7.241E-13 |
| AA_DQA1_187_32718380_A | 32718380 | 0.22 | 0.10 | 2.75 | 2.14 - 3.54 | 2.81E-15 | 7.241E-13 |
| AA_DQA1_187_32718380_T | 32718380 | 0.22 | 0.10 | 2.75 | 2.14 - 3.54 | 2.81E-15 | 7.241E-13 |
| HLA-DQB1*04:02 | 32739039 | 0.09 | 0.02 | 6.11 | 3.89 - 9.60 | 3.88E-15 | 9.402E-13 |
| AA_DRB1_67_32659947_L | 32659947 | 0.43 | 0.26 | 2.14 | 1.77 - 2.59 | 7.91E-15 | 1.811E-12 |
| AA_DQB1_185_32737733_I | 32737733 | 0.23 | 0.11 | 2.47 | 1.94 - 3.15 | 2.02E-13 | 4.27E-11 |
| AA_DRB1_231_32656010_P | 32656010 | 0.09 | 0.02 | 5.03 | 3.24 - 7.82 | 6.60E-13 | 1.111E-10 |
| AA_DRB1_166_32657380_Q | 32657380 | 0.09 | 0.02 | 5.03 | 3.24 - 7.82 | 6.60E-13 | 1.111E-10 |
| AA_DRB1_40_32660028 | 32660028 | 0.09 | 0.02 | 5.03 | 3.24 - 7.82 | 6.60E-13 | 1.111E-10 |
| AA_DRB1_38_32660034_A | 32660034 | 0.09 | 0.02 | 5.03 | 3.24 - 7.82 | 6.60E-13 | 1.111E-10 |
| HLA-DRB1*10:01 | 32660042 | 0.09 | 0.02 | 5.03 | 3.24 - 7.82 | 6.60E-13 | 1.111E-10 |
| AA_DRB1_31_32660055_V | 32660055 | 0.09 | 0.02 | 5.03 | 3.24 - 7.82 | 6.60E-13 | 1.111E-10 |
| AA_DRB1_30_32660058_R | 32660058 | 0.09 | 0.02 | 5.03 | 3.24 - 7.82 | 6.60E-13 | 1.111E-10 |
| AA_DRB1_10_32660118_E | 32660118 | 0.09 | 0.02 | 5.03 | 3.24 - 7.82 | 6.60E-13 | 1.111E-10 |
| AA_DQB1_74_32740615_S | 32740615 | 0.50 | 0.33 | 1.98 | 1.64 - 2.39 | 1.04E-12 | 1.677E-10 |
| AA_DQB1_26_32740759_G | 32740759 | 0.50 | 0.33 | 1.98 | 1.64 - 2.39 | 1.04E-12 | 1.677E-10 |
| AA_DRB1_149_32657431_H | 32657431 | 0.32 | 0.48 | 0.51 | 0.42 - 0.62 | 1.19E-11 | 1.75E-09 |
| AA_DRB1_12_32660112 | 32660112 | 0.32 | 0.48 | 0.51 | 0.42 - 0.62 | 1.39E-11 | 1.945E-09 |
| AA_DRB1_11_32660115_S | 32660115 | 0.32 | 0.48 | 0.51 | 0.42 - 0.62 | 1.39E-11 | 1.945E-09 |
| AA_DRB1_10_32660118_Y | 32660118 | 0.32 | 0.48 | 0.51 | 0.42 - 0.62 | 1.39E-11 | 1.945E-09 |
| AA_DRB1_47_32660007 | 32660007 | 0.51 | 0.36 | 1.87 | 1.55 - 2.25 | 4.60E-11 | 6.213E-09 |
| AA_DQA1_56_32717218_x | 32717218 | 0.31 | 0.45 | 0.53 | 0.43 - 0.64 | 2.53E-10 | 3.019E-08 |
| AA_DQA1_76_32717278_L | 32717278 | 0.31 | 0.45 | 0.53 | 0.43 - 0.64 | 2.53E-10 | 3.019E-08 |
| AA_DRB1_233_32656004_R | 32656004 | 0.31 | 0.45 | 0.53 | 0.44 - 0.65 | 3.26E-10 | 3.791E-08 |
| AA_DQB1_-18_32742319_V | 32742319 | 0.59 | 0.45 | 1.78 | 1.48 - 2.15 | 1.24E-09 | 1.349E-07 |
| AA_DQB1_-18_32742319_A | 32742319 | 0.60 | 0.46 | 1.77 | 1.47 - 2.13 | 2.31E-09 | 2.44E-07 |
| AA_DRB1_13_32660109_F | 32660109 | 0.13 | 0.06 | 2.45 | 1.81 - 3.32 | 7.13E-09 | 7.26E-07 |
| HLA-DQB1*04:01 | 32739039 | 0.06 | 0.01 | 4.83 | 2.82 - 8.28 | 1.03E-08 | 1.014E-06 |
| AA_DQB1_23_32740768 | 32740768 | 0.06 | 0.01 | 4.83 | 2.82 - 8.28 | 1.03E-08 | 1.014E-06 |
| AA_DQB1_-6_32742283_A | 32742283 | 0.06 | 0.01 | 4.83 | 2.82 - 8.28 | 1.03E-08 | 1.014E-06 |
| AA_DRB1_86_32659890 | 32659890 | 0.36 | 0.49 | 0.59 | 0.48 - 0.71 | 3.03E-08 | 2.941E-06 |
| **Chinese** | | | | | | | |
| HLA-DRB1*04:05 | 32660042 | 0.18 | 0.04 | 5.22 | 2.95 - 9.25 | 1.52E-08 | 0.0001254 |
| AA_DRB1_120_32657518_N | 32657518 | 0.28 | 0.12 | 3.05 | 2.03 - 4.58 | 8.43E-08 | 0.0001497 |
| AA_DQB1_9_32740810_F | 32740810 | 0.22 | 0.08 | 3.66 | 2.27 - 5.88 | 9.09E-08 | 0.0001497 |
| AA_DQB1_23_32740768 | 32740768 | 0.16 | 0.03 | 5.46 | 2.91 - 10.23 | 1.18E-07 | 0.0001497 |
| AA_DRB1_12_32660112 | 32660112 | 0.29 | 0.50 | 0.43 | 0.31 - 0.59 | 1.45E-07 | 0.0001497 |
| AA_DRB1_11_32660115_S | 32660115 | 0.29 | 0.50 | 0.43 | 0.31 - 0.59 | 1.45E-07 | 0.0001497 |
| AA_DRB1_10_32660118_Y | 32660118 | 0.29 | 0.50 | 0.43 | 0.31 - 0.59 | 1.45E-07 | 0.0001497 |
| HLA-DQB1*04:01 | 32739039 | 0.15 | 0.03 | 5.30 | 2.83 - 9.94 | 1.98E-07 | 0.0001628 |
| AA_DQB1_-6_32742283_A | 32742283 | 0.15 | 0.03 | 5.30 | 2.83 - 9.94 | 1.98E-07 | 0.0001628 |
| AA_DRB1_149_32657431_H | 32657431 | 0.30 | 0.49 | 0.44 | 0.32 - 0.60 | 2.61E-07 | 0.0001794 |
| AA_DRB1_11_32660115_V | 32660115 | 0.27 | 0.12 | 2.87 | 1.91 - 4.30 | 3.63E-07 | 0.0001882 |
| AA_DQB1_71_32740624_D | 32740624 | 0.17 | 0.05 | 4.09 | 2.37 - 7.06 | 4.11E-07 | 0.0001882 |
| AA_DQB1_70_32740627_E | 32740627 | 0.17 | 0.05 | 4.09 | 2.37 - 7.06 | 4.11E-07 | 0.0001882 |
| AA_DQB1_56_32740669 | 32740669 | 0.17 | 0.05 | 4.09 | 2.37 - 7.06 | 4.11E-07 | 0.0001882 |
| AA_DRB1_180_32657338_L | 32657338 | 0.25 | 0.11 | 2.64 | 1.75 - 3.98 | 3.97E-06 | 0.00109 |
| AA_DRB1_96_32657590_Y | 32657590 | 0.25 | 0.11 | 2.64 | 1.75 - 3.98 | 3.97E-06 | 0.00109 |
| AA_DRB1_-16_32665457_V | 32665457 | 0.33 | 0.50 | 0.49 | 0.36 - 0.67 | 5.47E-06 | 0.001185 |
| AA_DRB1_-16_32665457_A | 32665457 | 0.33 | 0.50 | 0.49 | 0.36 - 0.67 | 5.47E-06 | 0.001185 |
| AA_DRB1_-25_32665484_R | 32665484 | 0.33 | 0.50 | 0.49 | 0.36 - 0.67 | 5.47E-06 | 0.001185 |
| AA_DRB1_74_32659926_A | 32659926 | 0.35 | 0.52 | 0.51 | 0.38 - 0.69 | 1.02E-05 | 0.001714 |
| HLA-DQA1*03:01 | 32716284 | 0.42 | 0.26 | 2.03 | 1.48 - 2.79 | 1.06E-05 | 0.001714 |
| AA_DQA1_26_32717128 | 32717128 | 0.42 | 0.26 | 2.03 | 1.48 - 2.79 | 1.06E-05 | 0.001714 |
| AA_DQA1_47_32717191_Q | 32717191 | 0.42 | 0.26 | 2.03 | 1.48 - 2.79 | 1.06E-05 | 0.001714 |
| AA_DQA1_56_32717218_R | 32717218 | 0.42 | 0.26 | 2.03 | 1.48 - 2.79 | 1.06E-05 | 0.001714 |
| AA_DQA1_76_32717278_V | 32717278 | 0.42 | 0.26 | 2.03 | 1.48 - 2.79 | 1.06E-05 | 0.001714 |
| AA_DQA1_187_32718380_A | 32718380 | 0.42 | 0.26 | 2.03 | 1.48 - 2.79 | 1.06E-05 | 0.001714 |
| AA_DQA1_187_32718380_T | 32718380 | 0.42 | 0.26 | 2.03 | 1.48 - 2.79 | 1.06E-05 | 0.001714 |
| AA_DRB1_33_32660049 | 32660049 | 0.24 | 0.11 | 2.48 | 1.64 - 3.73 | 1.48E-05 | 0.002179 |
| AA_DRB1_13_32660109_H | 32660109 | 0.24 | 0.11 | 2.48 | 1.64 - 3.73 | 1.48E-05 | 0.002179 |
| AA_DRB1_-24_32665481_F | 32665481 | 0.24 | 0.11 | 2.48 | 1.64 - 3.73 | 1.48E-05 | 0.002179 |
| AA_DRB1_233_32656004_R | 32656004 | 0.25 | 0.41 | 0.50 | 0.36 - 0.69 | 1.95E-05 | 0.002674 |
| AA_DQB1_185_32737733_I | 32737733 | 0.42 | 0.27 | 1.97 | 1.43 - 2.71 | 2.86E-05 | 0.003571 |
| AA_DRB1_86_32659890 | 32659890 | 0.34 | 0.49 | 0.52 | 0.38 - 0.71 | 3.36E-05 | 0.004067 |
| AA_DQA1_50_32717200_L | 32717200 | 0.44 | 0.29 | 1.90 | 1.40 - 2.58 | 4.45E-05 | 0.004895 |
| AA_DQA1_53_32717209_R | 32717209 | 0.44 | 0.29 | 1.90 | 1.40 - 2.58 | 4.45E-05 | 0.004895 |
| AA_DQA1_215_32718464_L | 32718464 | 0.44 | 0.29 | 1.90 | 1.40 - 2.58 | 4.45E-05 | 0.004895 |
| AA_DRB1_104_32657566_A | 32657566 | 0.43 | 0.29 | 1.88 | 1.38 - 2.56 | 5.79E-05 | 0.006047 |
| AA_DRB1_98_32657584_E | 32657584 | 0.43 | 0.29 | 1.88 | 1.38 - 2.56 | 5.79E-05 | 0.006047 |
| AA_DRB1_32_32660052 | 32660052 | 0.22 | 0.36 | 0.52 | 0.37 - 0.72 | 0.000104 | 0.01032 |
| AA_DRB1_13_32660109_S | 32660109 | 0.13 | 0.24 | 0.47 | 0.32 - 0.70 | 0.000177 | 0.01618 |
| AA_DRB1_57_32659977_S | 32659977 | 0.22 | 0.12 | 2.14 | 1.43 - 3.20 | 0.000233 | 0.02058 |
| AA_DQB1_-10_32742295_A | 32742295 | 0.04 | 0.13 | 0.33 | 0.18 - 0.59 | 0.000243 | 0.0211 |
| AA_DRB1_37_32660037_Y | 32660037 | 0.35 | 0.23 | 1.79 | 1.29 - 2.46 | 0.000417 | 0.03241 |
| AA_DRB1_73_32659929 | 32659929 | 0.04 | 0.13 | 0.34 | 0.19 - 0.62 | 0.000429 | 0.03303 |
| HLA-DQB1*02:01 | 32739039 | 0.04 | 0.12 | 0.34 | 0.19 - 0.62 | 0.000471 | 0.03498 |
| AA_DQB1_-10_32742295_S | 32742295 | 0.04 | 0.12 | 0.34 | 0.19 - 0.62 | 0.000471 | 0.03498 |
| AA_DQA1_56_32717218_x | 32717218 | 0.20 | 0.32 | 0.55 | 0.39 - 0.77 | 0.000531 | 0.03619 |
| AA_DQA1_76_32717278_L | 32717278 | 0.20 | 0.32 | 0.55 | 0.39 - 0.77 | 0.000531 | 0.03619 |
| AA_DRB1_71_32659935_K | 32659935 | 0.03 | 0.11 | 0.31 | 0.16 - 0.6 | 0.000603 | 0.0398 |
| AA_DRB1_77_32659917 | 32659917 | 0.03 | 0.10 | 0.30 | 0.15 - 0.60 | 0.000695 | 0.04244 |
| AA_DRB1_74_32659926_R | 32659926 | 0.03 | 0.10 | 0.30 | 0.15 - 0.60 | 0.000695 | 0.04244 |
| AA_DRB1_60_32659968_H | 32659968 | 0.03 | 0.10 | 0.30 | 0.15 - 0.60 | 0.000695 | 0.04244 |
| AA_DRB1_57_32659977_A | 32659977 | 0.03 | 0.10 | 0.30 | 0.15 - 0.60 | 0.000695 | 0.04244 |
| HLA-DRB1*03:01 | 32660042 | 0.03 | 0.10 | 0.30 | 0.15 - 0.60 | 0.000695 | 0.04244 |
| AA_DQB1_74_32740615_A | 32740615 | 0.05 | 0.12 | 0.37 | 0.20 - 0.66 | 0.000777 | 0.04451 |
| AA_DQB1_71_32740624_K | 32740624 | 0.05 | 0.12 | 0.37 | 0.20 - 0.66 | 0.000777 | 0.04451 |
| AA_DQB1_55_32740672_L | 32740672 | 0.05 | 0.12 | 0.37 | 0.20 - 0.66 | 0.000777 | 0.04451 |
| AA_DQB1_52_32740681 | 32740681 | 0.05 | 0.12 | 0.37 | 0.20 - 0.66 | 0.000777 | 0.04451 |
| AA_DQB1_47_32740696 | 32740696 | 0.05 | 0.12 | 0.37 | 0.20 - 0.66 | 0.000777 | 0.04451 |
| AA_DQB1_46_32740699 | 32740699 | 0.05 | 0.12 | 0.37 | 0.20 - 0.66 | 0.000777 | 0.04451 |
| AA_DQB1_37_32740726_I | 32740726 | 0.05 | 0.12 | 0.37 | 0.20 - 0.66 | 0.000777 | 0.04451 |
| AA_DQB1_30_32740747_S | 32740747 | 0.05 | 0.12 | 0.37 | 0.20 - 0.66 | 0.000777 | 0.04451 |
| AA_DQB1_28_32740753 | 32740753 | 0.05 | 0.12 | 0.37 | 0.20 - 0.66 | 0.000777 | 0.04451 |
| **Indian** | | | | | | | |
| AA_DRB1_96_32657590_H | 32657590 | 0.37 | 0.53 | 0.48 | 0.37 - 0.62 | 2.58E-08 | 0.0002127 |
| AA_DRB1_13_32660109_F | 32660109 | 0.23 | 0.12 | 2.41 | 1.71 - 3.40 | 4.54E-07 | 0.0007952 |
| AA_DRB1_11_32660115_V | 32660115 | 0.35 | 0.22 | 1.99 | 1.52 - 2.61 | 6.60E-07 | 0.0008877 |
| AA_DRB1_120_32657518_N | 32657518 | 0.35 | 0.22 | 1.97 | 1.51 - 2.59 | 8.90E-07 | 0.0008877 |
| HLA-DQB1*05:01 | 32739039 | 0.22 | 0.11 | 2.39 | 1.69 - 3.39 | 9.69E-07 | 0.0008877 |
| AA_DRB1_149_32657431_H | 32657431 | 0.24 | 0.37 | 0.53 | 0.40 - 0.69 | 2.93E-06 | 0.0008969 |
| AA_DRB1_26_32660070_L | 32660070 | 0.25 | 0.14 | 2.13 | 1.54 - 2.93 | 4.21E-06 | 0.0008969 |
| AA_DRB1_231_32656010_P | 32656010 | 0.18 | 0.09 | 2.44 | 1.67 - 3.57 | 4.57E-06 | 0.0008969 |
| AA_DRB1_166_32657380_Q | 32657380 | 0.18 | 0.09 | 2.44 | 1.67 - 3.57 | 4.57E-06 | 0.0008969 |
| AA_DRB1_40_32660028 | 32660028 | 0.18 | 0.09 | 2.44 | 1.67 - 3.57 | 4.57E-06 | 0.0008969 |
| AA_DRB1_38_32660034_A | 32660034 | 0.18 | 0.09 | 2.44 | 1.67 - 3.57 | 4.57E-06 | 0.0008969 |
| HLA-DRB1*10:01 | 32660042 | 0.18 | 0.09 | 2.44 | 1.67 - 3.57 | 4.57E-06 | 0.0008969 |
| AA_DRB1_31_32660055_V | 32660055 | 0.18 | 0.09 | 2.44 | 1.67 - 3.57 | 4.57E-06 | 0.0008969 |
| AA_DRB1_30_32660058_R | 32660058 | 0.18 | 0.09 | 2.44 | 1.67 - 3.57 | 4.57E-06 | 0.0008969 |
| AA_DRB1_10_32660118_E | 32660118 | 0.18 | 0.09 | 2.44 | 1.67 - 3.57 | 4.57E-06 | 0.0008969 |
| AA_DRB1_233_32656004_R | 32656004 | 0.22 | 0.35 | 0.53 | 0.41 - 0.7 | 5.71E-06 | 0.00107 |
| AA_DRB1_12_32660112 | 32660112 | 0.24 | 0.37 | 0.54 | 0.41 - 0.71 | 7.18E-06 | 0.00126 |
| AA_DRB1_11_32660115_S | 32660115 | 0.24 | 0.37 | 0.54 | 0.41 - 0.71 | 7.18E-06 | 0.00126 |
| AA_DRB1_10_32660118_Y | 32660118 | 0.24 | 0.37 | 0.54 | 0.41 - 0.71 | 7.18E-06 | 0.00126 |
| AA_B_24_31432644_S | 31432644 | 0.31 | 0.21 | 1.77 | 1.35 - 2.34 | 4.52E-05 | 0.004384 |
| AA_A_166_30019249 | 30019249 | 0.28 | 0.39 | 0.60 | 0.46 - 0.77 | 7.05E-05 | 0.006128 |
| AA_A_167_30019252 | 30019252 | 0.28 | 0.39 | 0.60 | 0.46 - 0.77 | 7.05E-05 | 0.006128 |
| AA_DRB1_70_32659938_D | 32659938 | 0.25 | 0.36 | 0.59 | 0.46 - 0.77 | 7.73E-05 | 0.006639 |
| AA_DQB1_57_32740666_V | 32740666 | 0.23 | 0.14 | 1.88 | 1.37 - 2.59 | 9.73E-05 | 0.008025 |
| AA_DRB1_37_32660037_Y | 32660037 | 0.39 | 0.28 | 1.64 | 1.28 - 2.11 | 0.000102 | 0.008319 |
| AA_DRB1_96_32657590_Q | 32657590 | 0.42 | 0.31 | 1.62 | 1.26 - 2.09 | 0.000213 | 0.01461 |
| AA_DRB1_67_32659947_L | 32659947 | 0.53 | 0.42 | 1.58 | 1.23 - 2.01 | 0.000262 | 0.01524 |
| AA_DRB1_13_32660109_S | 32660109 | 0.12 | 0.20 | 0.56 | 0.41 - 0.77 | 0.000394 | 0.01899 |
| AA_B_24_31432644_A | 31432644 | 0.42 | 0.53 | 0.66 | 0.52 - 0.83 | 0.00041 | 0.01967 |
| AA_C_156_31346909_L | 31346909 | 0.48 | 0.37 | 1.54 | 1.21 - 1.95 | 0.000435 | 0.02058 |
| AA_DRB1_71_32659935_E | 32659935 | 0.04 | 0.10 | 0.42 | 0.26 - 0.69 | 0.000539 | 0.02476 |
| AA_A_76_30018738_E | 30018738 | 0.16 | 0.24 | 0.59 | 0.44 - 0.80 | 0.000626 | 0.02647 |
| AA_A_79_30018747 | 30018747 | 0.16 | 0.24 | 0.59 | 0.44 - 0.80 | 0.000626 | 0.02647 |
| AA_A_80_30018750 | 30018750 | 0.16 | 0.24 | 0.59 | 0.44 - 0.80 | 0.000626 | 0.02647 |
| AA_A_82_30018756 | 30018756 | 0.16 | 0.24 | 0.59 | 0.44 - 0.80 | 0.000626 | 0.02647 |
| AA_A_83_30018759 | 30018759 | 0.16 | 0.24 | 0.59 | 0.44 - 0.80 | 0.000626 | 0.02647 |
| AA_C_97_31347086 | 31347086 | 0.28 | 0.38 | 0.65 | 0.51 - 0.84 | 0.000749 | 0.03102 |
| AA_A_77_30018741_N | 30018741 | 0.32 | 0.42 | 0.66 | 0.52 - 0.84 | 0.000786 | 0.03193 |
| AA_DQA1_25_32717125 | 32717125 | 0.33 | 0.42 | 0.66 | 0.52 - 0.84 | 0.000883 | 0.0357 |
| AA_C_194_31346208_L | 31346208 | 0.29 | 0.20 | 1.59 | 1.20 - 2.10 | 0.001127 | 0.04284 |
| AA_C_184_31346238_P | 31346238 | 0.29 | 0.20 | 1.59 | 1.20 - 2.10 | 0.001127 | 0.04284 |
| AA_C_147_31346936 | 31346936 | 0.29 | 0.20 | 1.59 | 1.20 - 2.10 | 0.001127 | 0.04284 |
| AA_DRB1_74_32659926_A | 32659926 | 0.36 | 0.46 | 0.67 | 0.53 - 0.86 | 0.001268 | 0.0469 |
| AA_DQB1_74_32740615_S | 32740615 | 0.36 | 0.27 | 1.53 | 1.18 - 1.97 | 0.001289 | 0.04715 |
| AA_DQB1_26_32740759_G | 32740759 | 0.36 | 0.27 | 1.53 | 1.18 - 1.97 | 0.001289 | 0.04715 |
| HLA-B*57:01 | 31431272 | 0.04 | 0.09 | 0.44 | 0.26 - 0.73 | 0.001418 | 0.04974 |
| AA_B_97_31432180_V | 31432180 | 0.04 | 0.09 | 0.44 | 0.26 - 0.73 | 0.001418 | 0.04974 |

**Footnote:** Imputed HLA variants nomenclature: (a) HLA amino acids: AA_[Gene]_[Amino Acid Position]_[Amino Acid Residue], (b) Classical HLA alleles: HLA-[Gene]*[Four-digit alleles]; ACPA: anti-citrullinated peptide autoantibodies; HLA: Human-leukocyte antigen; Position: base-pair position of SNP, OR: odds-ratio, 95% CI: 95% confidence interval, P: p-value; FDR-BH: false discovery rate by Benjamin&Hochberg. The HLA variants were arranged in the ascending order of p-value.

**Supplementary table s6** Stepwise logistic regression analysis for risk of ACPA-positive RA in the Malay ethnic group

|  | **HLA variants** | **Odds ratio (OR)** | **95% confidence interval** | **p-value** |
| --- | --- | --- | --- | --- |
| Step 1^a^ | HLA-DRB1 Val11^#^ | 2.896 | 2.31 – 3.64 | 4.73x10^-20^ |
| Step 2^b^ | *HLA-DRB1*12:02* | 0.382 | 0.28 – 0.52 | 2.45 x10^-10^ |
|  | HLA-DRB1 Val11^#^ | 2.569 | 2.04 – 3.24 | 1.01 x10^-15^ |
| Step 3^c^ | *HLA-DQB1*03:02* | 0.273 | 0.17 – 0.45 | 2.30 x10^-07^ |
|  | *HLA-DRB1*12:02* | 0.377 | 0.28 – 0.51 | 1.79 x10^-10^ |
|  | HLA-DRB1 Val11 | 3.492 | 2.68 – 4.56 | 2.80 x10^-20^ |

^a^:Variable entered on step 1: HLA-DRB1 Val11, ^b^:Variables entered on step 2: *HLA-DRB1*1202,* ^c^:Variables entered on step 3: *HLA-DQB1*0302*. ^#^Individuals carrying *HLA-DRB1*04:01, *04:02, *04:03, *04:04, *04:05, *04:06, *04:10,* and **10:01* alleles were defined as positive HLA-DRB1 Val11.

| a) |  | b) | 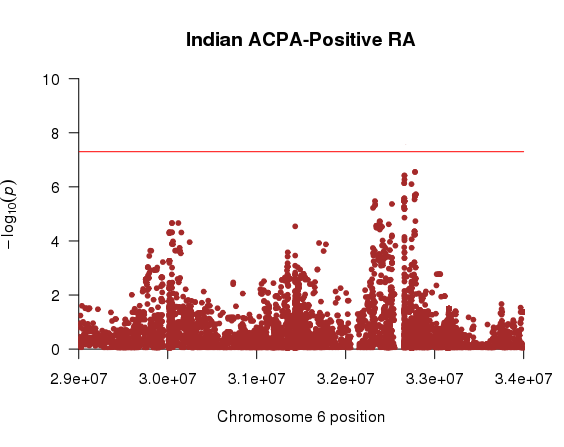 |
| --- | --- | --- | --- |

**Supplementary figure 1. Plot of stepwise logistic regression analysis to fine-map HLA variants as risk factor for ACPA-positive RA in the Chinese and Indian ethnic groups**. a) Regional plot of stepwise logistic regression in ACPA-positive Chinese RA patients. Upon conditioning of *HLA-DRB1*04:05* allele using stepwise logistic regression analysis showed no significant independent association between other HLA variants and risk of developing ACPA-positive RA in in the Chinese ethnic groups. b) Regional plot of stepwise logistic regression in ACPA-positive Indian RA patients. Conditioning on HLA-DRB1 His96 variant using stepwise logistic regression analysis showed no significant independent association of other HLA variants from HLA-DRB1 His96 variant, and risk of developing ACPA-positive RA in in the Indian ethnic groups. The red line represents the genome-wide significant threshold of p<5x10^-8^.


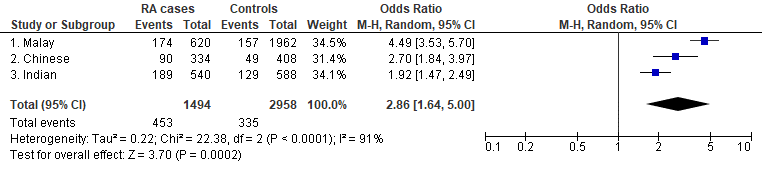


**(a)**


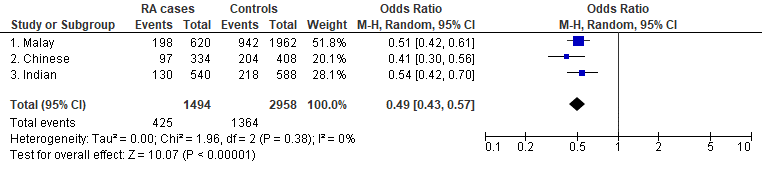


**(b)**


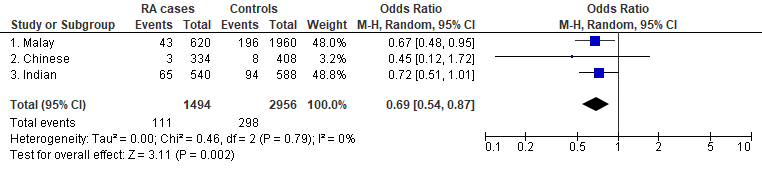


**(c)**


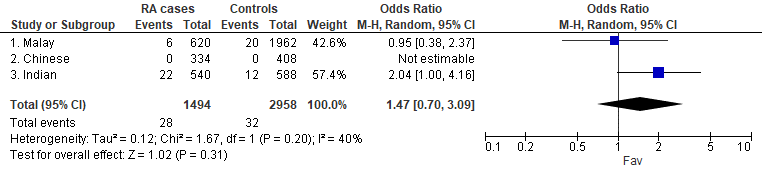


**(d)**


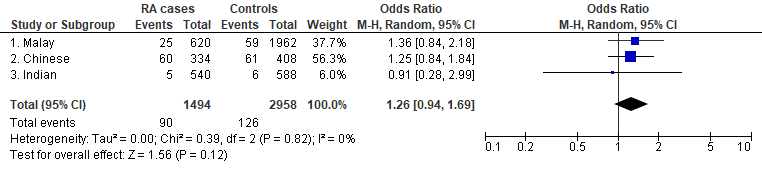


**(e)**


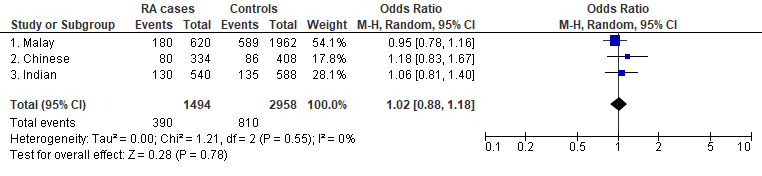


**(f)**

**Supplementary figure 2 Meta-analysis of polymorphic HLA-DRB1 amino acid residues position 11 and risk of developing ACPA-positive RA in Malay, Chinese and Indian ethnic groups**. (a) valine residue (b) serine residue, (c) glycine residue (d) leucine residue (e) aspartic residue (f) proline residue. *HLA-DRB1*: HLA DR beta 1 gene; ACPA: anti-citrullinated peptide antibody; RA: rheumatoid arthritis; P=p-value; OR=odds ratio; *I^2^*: heterogeneity
